# SpaBiT: enhancing spatial transcriptomics resolution via bidirectional attention transformers

**DOI:** 10.1093/bioinformatics/btag443

**Published:** 2026-07-02

**Authors:** Xiaofei Liu, Ao Li, Wenwen Min

**Affiliations:** School of Information Science and Engineering, Yunnan University, Yunnan 650500, China; School of Information Science and Engineering, Yunnan University, Yunnan 650500, China; School of Information Science and Engineering, Yunnan University, Yunnan 650500, China

## Abstract

**Motivation:**

Spatial transcriptomics (STs) enables the precise mapping of gene expression within tissue architecture, however its application is often limited by low spatial resolution and sparse sampling. While existing deep learning methods leverage histology images, spatial coordinates, or low-resolution expression data to predict high-density profiles, these methods are limited in either capturing the intrinsic constraints between histological context and spatial topology or ignoring the complex local neighborhood relationships between spots.

**Results:**

To address these limitations, we propose SpaBiT, a multimodal framework designed to enhance ST resolution via a bidirectional attention mechanism. At its core, SpaBiT employs a bidirectional cross-attention module to facilitate precise information exchange between image features and neighborhood-aware representations learned via a graph attention network. This design explicitly models the synergistic constraints between local morphology and spatial graph topology, yielding high-fidelity, high-density gene expression maps. SpaBiT exhibits competitive performance in reconstructing complex spatial gene expression, outperforming the benchmark models utilized in this study across various quantitative metrics, providing a robust tool for deciphering complex tissue microenvironments.

**Availability and implementation:**

The source code and datasets are available at https://github.com/wenwenmin/SpaBiT.

## 1 Introduction

The spatial information of gene expression in complex tissues can help to depict the spatial distribution of various cell types (Zhang *et al.* 2024c) and enable the understanding of key biological processes such as the tumor microenvironment (Ji *et al.* 2020, Zheng *et al.* 2023), organ development (Asp *et al.* 2019), disease mechanism (Chen *et al.* 2020), and regeneration mechanism (Cui *et al.* 2023), and spatial domain identification for tissue heterogeneity dissection via structure correlation and self-representation (Zhang *et al.* 2024b) or multi-graph fusion (Ma *et al.* 2026). Recent developments in spatial transcriptomics (STs) capture spatial information by using DNA barcodes to distinguish different spots in the tissue (Ståhl *et al.* 2016).

10x Genomics Visium (Janesick *et al.* 2023) is a widely used ST technology, where the center-to-center distance between neighboring capture spots is approximately 100 μm and each spot has a diameter of approximately 55 μm. This implies that only a fraction of the tissue surface is actually covered by capture spots. Ji *et al.* (2020) presented that only about one-third of the tissue section can produce gene expression data. Those unmeasured regions, such as tumor margins, often harbor critical disease signals (Zhao *et al.* 2021). However, experimental resolution enhancement is costly and time-consuming for large-scale studies (Min *et al.* 2024). Therefore, cost-effective methods for enhancing STs resolution and addressing sparse spot distribution are essential for advancing ST technology.

Some recent methods have been developed to enhance STs resolution. Both ST-Net (He *et al.* 2020) and DeepSpaCE (Monjo *et al.* 2022) are approaches based on convolutional neural networks using histology images. Specifically, ST-Net uses DenseNet-121 as the backbone and DeepSpaCE is built upon a VGG16 architecture. STAGE (Li *et al.* 2024) focuses on spatial information by integrating spot coordinates and gene expression into a spatially supervised autoencoder, which reconstructs low-dimensional coordinates to predict gene expression at unmeasured locations.

HisToSGE (Shi *et al.* 2024) innovatively integrates a pathology foundation model [UNI (Chen *et al.* 2024)] with spatial positional encodings. However, its spatial modeling relies primarily on global absolute coordinates, which tends to treat individual capture spots as isolated entities and presents difficulties in explicitly capturing the continuous, local geometric neighborhoods, and spatial topologies. Furthermore, within its fusion mechanism, the histological features and global coordinate encodings are combined via element-wise addition prior to the standard Transformer. This integration to some extent treats spatial information as a static spatial bias, leaving room for further exploration of deep, non-linear local interactions between the multimodal modalities.

Both DIST (Zhao *et al.* 2023) and SpaViT (Min *et al.* 2026) are approaches using the original low-resolution gene expression data. Specifically, DIST uses convolutional neural networks and SpaViT uses Vision Transformer (Han *et al.* 2023). Note that the performance of DIST can be constrained when the expression measurements are highly noisy or extremely sparse, and the performance of SpaViT can be constrained in complex tissues with strong heterogeneity. While these models demonstrate robust performance across various applications, they may not fully account for the intrinsic constraints between histology context and spatial topology. As a result, accurate prediction of gene expression in highly heterogeneous tissues remains a challenge.

In response to these challenges, we propose SpaBiT, a multimodal framework that integrates histology images with STs. Specifically, SpaBiT employs a bidirectional cross-attention (CA) module to capture the intrinsic constraints between histology context and spatial topology. Local multimodal contexts are effectively encoded within SpaBiT by intertwining UNI-extracted morphological features with spatial neighborhood features learned via a graph attention network. Subsequently, a Transformer is integrated to capture global spatial dependencies, yielding high-density gene expression predictions. Our main contributions can be summarized as follows:

• We introduce a novel bidirectional CA mechanism that explicitly models the intrinsic constraints between histology context and neighborhood-aware spatial features, enabling precise encoding of local multimodal information.
• Evaluated across multiple ST platforms, SpaBiT consistently outperforms existing baselines in prediction accuracy [e.g., Pearson Correlation Coefficient (PCC)] and the reconstruction of complex spatial gene expression patterns.
• The high-density expression maps generated by SpaBiT preserve and strengthen biologically meaningful spatial signals, facilitating spatial domain identification and functional enrichment analyses in complex tissue microenvironments.

## 2 Materials and methods

### 2.1 Overview of the SpaBiT

**Figure 1:**
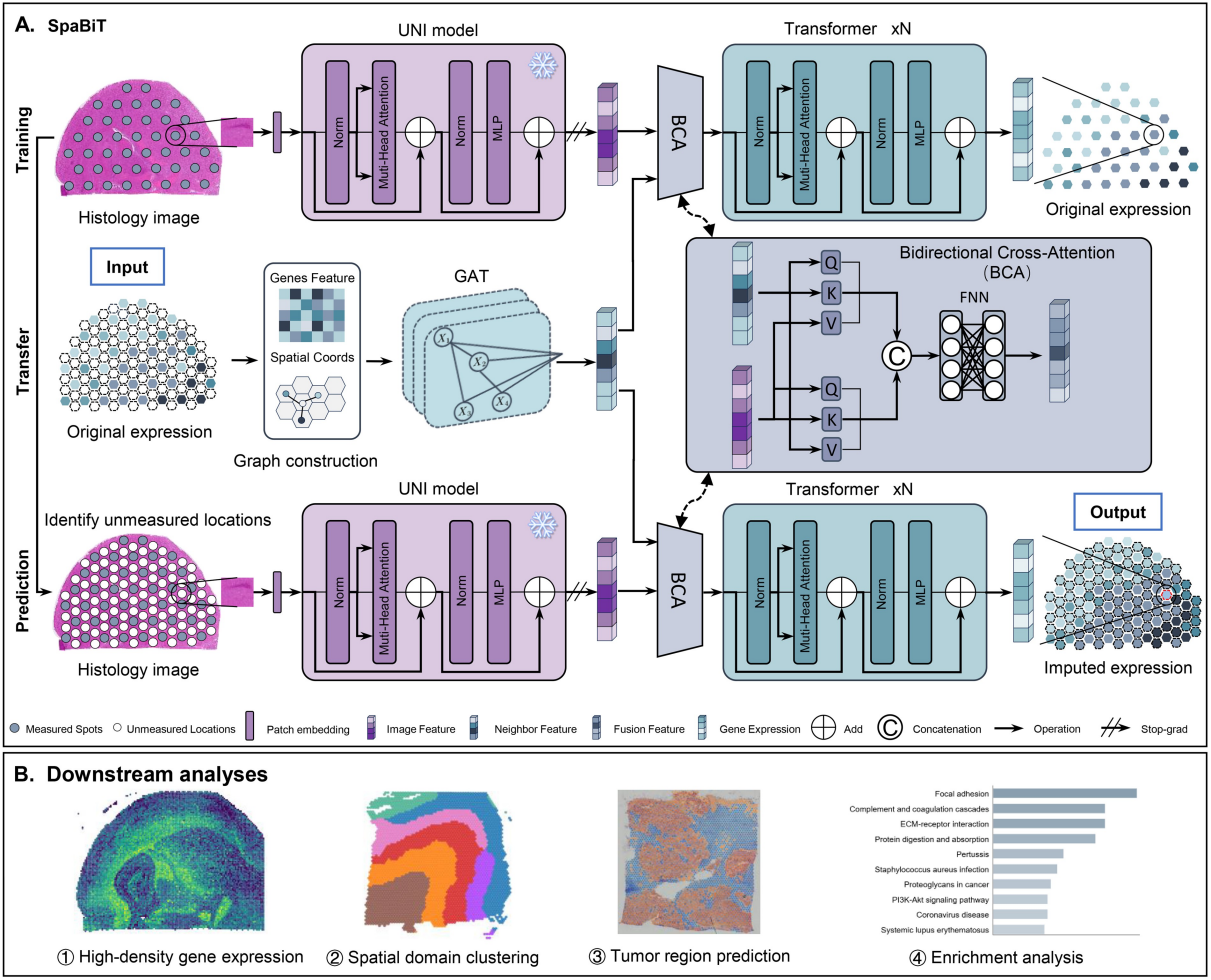
Overview of SpaBiT. (a) SpaBiT extracts histology features via the UNI foundation model and captures neighborhood expression via a GAT. These modalities are integrated through bidirectional cross-attention and processed by a Transformer to generate high-density spatial gene expression profiles. (b) Downstream analyses.

SpaBiT is a hierarchical multimodal framework designed for high-resolution gene expression reconstruction, as illustrated in Fig. 1. The input to our model consists of histology images and raw STs data. During the training process, the model is supervised using the measured spots. Specifically, we first capture image features from patch embeddings via UNI. Then we construct a spatial spot graph based on both the original gene expression and spatial coordinates. While neighbor features are generated by a Graph Attention Network [GAT] (Ye and Ji 2023) over the established spatial spot graph. Both feature sets are integrated through a bidirectional CA module to facilitate fine-grained modality interactions. Subsequently, a Transformer layer captures global spatial dependencies to refine the multimodal features. During the prediction phase, the trained model is applied to unmeasured locations to generate high-resolution impute expression predictions.

### 2.2 Data and data pre-processing

We evaluated our method on a diverse collection of STs datasets spanning multiple tissues, species, and sequencing platforms (Table 1). Specifically, the datasets include human dorsolateral prefrontal cortex (DLPFC) with 12 slices, as well as multiple single-slice datasets covering both normal and diseased tissues, such as mouse brain sagittal posterior (MBSP), human cervical cancer (HCC), human intestine cancer (HIC), human breast cancer (BC), human squamous cell carcinoma (HS), and HER2-positive breast tumor (E1). To further assess cross-platform generalization, we additionally incorporated datasets generated from different ST technologies, including 10× Visium, original STs, STARmap, Visium HD, and Xenium platforms. These datasets also include mouse placenta (MP), high-resolution mouse brain (MBHD), high-resolution human breast cancer (HBCHD), and two human liver tissue sections (HL1 and HL2).

**Table 1:** Summary of the ST datasets used in materials in this study.

Datasets	Tissue	Slice number	Platform
DLPFC (Maynard *et al.* 2021)	Human Dorsolateral Prefrontal Cortex	12	10x Visium
MBSP (Janesick *et al.* 2023)	Mouse brain sagittal posterior	1	10x Visium
HCC (Janesick *et al.* 2023)	Human cervical cancer	1	10x Visium
HIC (Janesick *et al.* 2023)	Human intestine cancer	1	10x Visium
BC (Shi *et al.* 2024)	Human breast cancer	1	Visium
HS (Ji *et al.* 2020)	Human Squamous Cell Carcinoma	1	ST
E1 (Andersson *et al.* 2021)	Human breast cancer	1	ST
MP (Thrane *et al.* 2018)	Mouse placenta	1	STARmap
MBHD (Janesick *et al.* 2023)	Mouse brain HD	1	Visium HD
HBCHD (Janesick *et al.* 2023)	Human breast cancer HD	1	Visium HD
HL1 (Janesick *et al.* 2023)	Human liver tissue	1	Xenium
HL2 (Janesick *et al.* 2023)	Human liver tissue	1	Xenium

Formally, given any tissue section, without loss of generality, we assume that this tissue section containing *N* spots, and denoted by {1,2,…,N}. Each spot i∈{1,…,N} is represented as a tuple $p_{i}=(x_{i},s_{i})$, where $x_{i}\in R^{G}$ and $s_{i}=(x_{i},y_{i})$ denote the gene expression vector of *G* selected genes and the spatial coordinates of spot *i* on this tissue section. To prepare the multimodal inputs, we first perform gene-level normalization. The top 300 highly variable genes are selected, followed by library size normalization and log-transformation to mitigate technical noise. Simultaneously, for the image modality, we segment the histology section into image patches of size 224×224 pixels. Each patch is centered at the corresponding spot coordinate $s_{i}$, capturing the local morphological context.

To evaluate SpaBiT’s performance in high-density reconstruction, we implement a structured down-sampling strategy that partitions the total set of *N* spots into an observed training set T (*Measured spots*) and a prediction set P (*Unmeasured locations*), such that T∪P={1,…,N} and T∩P=∅.

• *Training and Evaluation Phase:* We sub-sample spots from the original spatial grid to create a regular lattice. In this phase, the retained lattice spots are designated as Measured spots (T), while the remaining in-tissue spots are defined as Unmeasured locations (P). This setup allows the use of original expression profiles in P as ground-truth for validating the model’s reconstruction accuracy.
• *Final Inference Phase:* To generate high-density expression maps for the entire tissue, all physically sampled spots on the platform serve as Measured spots (T), while the target high-resolution grid points are designated as the actual Unmeasured locations (P).

Detailed down-sampling schemes are further provided in Supplementary Materials S1, available as supplementary data at *Bioinformatics* online.

### 2.3 SpaBiT model

#### 2.3.1 Histology image feature encoding via UNI

For each tissue section, based on the image patches provided in the pre-processing stage, we associate each spot i∈{1,…,N} with its local visual context. Formally, let ${\text{Patch}}_{i}\in R^{W\times H\times 3}$ denote the image patch centered at spot coordinate $s_{i}$. To extract representative morphological features, we employ a pre-trained UNI model as a feature extractor. Each patch is mapped to a high-dimensional embedding space to obtain the image feature:


(1) $$h_{i}^{\text{img}}=\text{UNI}({\text{Patch}}_{i}),\;h_{i}^{\text{img}}\in R^{d},$$

where $h_{i}^{\text{img}}$ represents the image feature of spot *i*, and *d* is the feature dimensionality. These image features serve as the visual modality inputs for subsequent multimodal fusion.

#### 2.3.2 Graph construction and neighborhood feature learning with GAT

Before constructing the spatial spot graph, we initialize the gene expression features for all spots. To prevent data leakage during training and ensure model generalization capability, it is critical that the model remains blind to the ground-truth expression of spots in the prediction set P (Unmeasured locations). Consequently, we construct a pre-filled expression matrix $\tilde{X}\in R^{N\times G}$ to serve as the initial state.

For observed spots in the training set (i∈T), we directly retain their original expression vectors:


(2) $${\tilde{x}}_{i}=x_{i},\;i\in T.$$

For spots in the prediction set (j∈P), we approximate their initial features by averaging the expressions of their *K* nearest observed neighbors within the coordinate space:


(3) $${\tilde{x}}_{j}=\frac{1}{K}\sum_{i\in N_{K}^{T}(j)}x_{i},\;j\in P,$$

where $N_{K}^{T}(j)$ denotes the set of *K* nearest neighbors of spot *j* within the observed set T. This yields a complete pre-filled matrix $\tilde{X}$ covering all *N* spots.

Based on the spatial coordinates ${\left\{s_{i}\right\}}_{i=1}^{N}$, we then construct a K-nearest-neighbor graph over all spots, resulting in an undirected adjacency matrix $A\in R^{N\times N}$. To capture local spatial dependencies, we apply a GAT (Ye and Ji 2023) to learn neighborhood-aware embeddings. Using the pre-filled vectors ${\tilde{x}}_{i}$ as initial node features, the attention-based aggregation for spot *i* is formulated as:


(4) $$h_{i}^{\text{nbr}}=\sigma \left(\sum_{j\in N(i)}\alpha_{ij}W{\tilde{x}}_{j}\right),$$

where N(i) is the neighbor set of node *i*, *W* is a learnable weight matrix, $\alpha_{ij}$ is the attention coefficient, and σ(·) is a non-linear activation function. The resulting vector $h_{i}^{\text{nbr}}$ is designated as the *neighbor feature* of spot *i*, effectively capturing the local spatial gene expression context.

#### 2.3.3 Bidirectional CA fusion and transformer-based prediction

After obtaining the image feature and neighborhood feature for each spot, BCA performs multimodal fusion at the spot level and predicts gene expression based on the fused representations. We first arrange the spot-wise features into two sequences:


(5) $$H^{\text{img}}=[h_{1}^{\text{img}},\ldots ,h_{N}^{\text{img}}],\;H^{\text{nbr}}=[h_{1}^{\text{nbr}},\ldots ,h_{N}^{\text{nbr}}],$$

which are used as inputs to the bidirectional CA module. We adopt a bidirectional CA mechanism composed of two parallel CA layers, where the basic CA operation is defined as:


(6) $$\text{CA}(Q,K,V)=\text{softmax}\left(\frac{QK^{⊤}}{\sqrt{d_{h}}}\right)V,$$

with *Q*, *K*, and *V* denoting the query, key, and value matrices, and $d_{h}$ the dimension of each attention head.

Specifically, we update the image features by attending to neighbor features and update the neighbor features by attending to image features:


(7) $$\begin{array}{c} Z_{1}=\text{CA}(H^{\text{img}},H^{\text{nbr}},H^{\text{nbr}}),\\ Z_{2}=\text{CA}(H^{\text{nbr}},H^{\text{img}},H^{\text{img}}). \end{array}$$

For each spot *i*, we then concatenate the corresponding vectors from $Z_{1}$ and $Z_{2}$ and feed them into a feed-forward neural network to obtain the fused multimodal feature:


(8) $$h_{i}^{\text{fusion}}=\text{FNN}(z_{1,i}⊕z_{2,i}),$$

where $z_{1,i}$ and $z_{2,i}$ are the *i*-th rows of $Z_{1}$ and $Z_{2}$, respectively. Collecting all spots yields the fused sequence:


(9) $$H^{\text{fusion}}=[h_{1}^{\text{fusion}},\ldots ,h_{N}^{\text{fusion}}].$$

The fused sequence $H^{\text{fusion}}$ is then fed into a Transformer encoder to model long-range dependencies among spots on the tissue. For each spot *i*, the corresponding Transformer output $h_{i}^{\text{trans}}$ is passed through a multilayer perceptron to obtain the predicted gene expression vector:


(10) $${\hat{x}}_{i}=\text{MLP}(h_{i}^{\text{trans}}),$$

where ${\hat{x}}_{i}\in R^{G}$ is the predicted expression profile of spot *i* over the selected *G* genes.

The loss function of SpaBiT is designed to minimize the discrepancy between the predicted and observed gene expression at the training spots, thereby encouraging accurate reconstruction of spatial gene expression patterns from multimodal inputs:


(11) $$L=\frac{1}{\left|T\right|}\sum_{i\in T}\|{\hat{x}}_{i}-x_{i}{\|}_{2}^{2},$$

where T denotes the index set of observed (training) spots, and $x_{i}$ and ${\hat{x}}_{i}$ are the observed and predicted gene expression vectors at spot *i*, respectively.

## 3 Results

### 3.1 Implementation details

For all baseline methods, we adopted the default parameters as specified in their respective original papers. Our experiments were conducted on a single NVIDIA RTX 4090 GPU. The training process was configured for 1000 epochs with a batch size of 128 and a learning rate of 0.001. More detailed model parameters are provided in the Supplementary Materials S2, available as supplementary data at *Bioinformatics* online.

### 3.2 Baseline methods

We compared our proposed model, SpaBiT, against six state-of-the-art baseline methods and commonly used interpolation approaches. Specifically, the evaluated methods include image feature-based approaches such as ST-Net (He *et al.* 2020), DeepSpaCE (Monjo *et al.* 2022), and HisToSGE (Shi *et al.* 2024); a spatial topology-based method, STAGE (Li *et al.* 2024), which leverages spatial positional encoding to extract features; gene expression graph-based methods, including SpaViT (Min *et al.* 2026) and DIST (Zhao *et al.* 2023), which enhance resolution by training on gene expression graphs; as well as classical interpolation methods, namely nearest-neighbor (NN), linear interpolation, and cubic spline interpolation. Detailed descriptions of the baselines are provided in Supplementary Materials S3, available as supplementary data at *Bioinformatics* online, and the definitions of the evaluation metrics are provided in Supplementary Materials S4, available as supplementary data at *Bioinformatics* online.

### 3.3 SpaBiT enables more accurate generation of high-density gene expression

In this study, we evaluate SpaBiT through two complementary experiments: (i) a reconstruction experiment on standard ST datasets and (ii) simulation experiments using the newly released Visium HD and Xenium platforms. For the reconstruction setting, we generate controlled low-resolution inputs by applying structured spatial downsampling that preserves the geometric configuration of each dataset. This yields a realistic pre-training setup with pseudo-missing regions and enables accurate quantification of reconstruction performance across unobserved areas. For the simulation experiments, we leverage high-resolution STs provided by Visium HD and Xenium. By aligning their high-density expression grids with conventional ST layouts, we construct pseudo low-resolution Visium inputs and corresponding high-resolution ground truth, the latter excluded entirely from model training to ensure fair evaluation. Through this design, we are able to directly evaluate SpaBiT’s ability to recover high-density spatial gene expression patterns from conventional spot-based ST data. To evaluate reconstruction accuracy, we used PCC, MSE, and MAE, and performed comparisons across 21 slices from 10 datasets covering multiple platforms.

As shown in Tables 2 and 3, SpaBiT achieves the highest PCC across all DLPFC slices, For MSE and MAE, SpaBiT achieves leading performance on most DLPFC slices. Across the remaining datasets, SpaBiT also shows stable and strong performance. For a more comprehensive evaluation, extended Jensen-Shannon divergence results on the DLPFC dataset are provided in Supplementary Materials S5, available as supplementary data at *Bioinformatics* online.

**Table 2:** Comparison of the performance of data generated by SpaBiT and baseline methods with the original data across three evaluation metrics on the DLPFC 12-slice dataset.

Slices	Pearson Correlation Coefficient (PCC)									
	SpaBiT	STAGE	HisToSGE	SpaViT	DIST	DeepSpaCE	ST-Net	NN	Linear	Cubic
#151507	**0.2867 ** ± ** 0.0122**	0.1893 ± 0.0016	0.1574 ± 0.0094	0.2194 ± 0.0124	0.2113 ± 0.0093	0.2048 ± 0.0073	0.1994 ± 0.0064	0.1311 ± 0.0000	0.2015 ± 0.0000	0.2016 ± 0.0000
#151508	**0.2423 ** ± ** 0.0449**	0.1930 ± 0.0046	0.1504 ± 0.0196	0.1940 ± 0.0104	0.1761 ± 0.0098	0.2074 ± 0.0073	0.2133 ± 0.0079	0.1288 ± 0.0000	0.2010 ± 0.0000	0.2011 ± 0.0000
#151509	**0.3196 ** ± ** 0.0060**	0.2265 ± 0.0041	0.1155 ± 0.0156	0.2278 ± 0.0112	0.2164 ± 0.0103	0.2194 ± 0.0046	0.2373 ± 0.0089	0.1457 ± 0.0000	0.2264 ± 0.0000	0.2265 ± 0.0000
#151510	**0.2710 ** ± ** 0.0061**	0.1819 ± 0.0023	0.0941 ± 0.0122	0.1803 ± 0.0134	0.1731 ± 0.0091	0.1841 ± 0.0096	0.1742 ± 0.0022	0.1176 ± 0.0000	0.1856 ± 0.0000	0.1856 ± 0.0000
#151669	**0.3366 ** ± ** 0.0481**	0.2445 ± 0.0030	0.1854 ± 0.0236	0.2316 ± 0.0154	0.2113 ± 0.0128	0.1928 ± 0.0284	0.2647 ± 0.0095	0.1774 ± 0.0000	0.2708 ± 0.0000	0.2708 ± 0.0000
#151670	**0.3283 ** ± ** 0.0591**	0.2404 ± 0.0034	0.1635 ± 0.0301	0.2205 ± 0.0164	0.2504 ± 0.0179	0.2458 ± 0.0284	0.2590 ± 0.0059	0.1641 ± 0.0000	0.2581 ± 0.0000	0.2581 ± 0.0000
#151671	**0.3722 ** ± ** 0.0386**	0.2642 ± 0.0029	0.1325 ± 0.0657	0.2638 ± 0.0185	0.3161 ± 0.0392	0.2776 ± 0.0109	0.2990 ± 0.0118	0.1872 ± 0.0000	0.2879 ± 0.0000	0.2880 ± 0.0000
#151672	**0.3350 ** ± ** 0.0142**	0.2408 ± 0.0019	0.1224 ± 0.0290	0.2594 ± 0.0294	0.2524 ± 0.0362	0.2279 ± 0.0243	0.2561 ± 0.0066	0.1677 ± 0.0000	0.2550 ± 0.0000	0.2550 ± 0.0000
#151673	**0.3965 ** ± ** 0.0210**	0.2932 ± 0.0025	0.1200 ± 0.0232	0.3464 ± 0.0084	0.3561 ± 0.0127	0.3153 ± 0.0101	0.3163 ± 0.0056	0.2216 ± 0.0000	0.3253 ± 0.0000	0.3253 ± 0.0000
#151674	**0.3662 ** ± ** 0.0608**	0.2499 ± 0.1267	0.1574 ± 0.0332	0.3488 ± 0.0237	0.3312 ± 0.0172	0.3160 ± 0.0104	0.3394 ± 0.0068	0.2203 ± 0.0000	0.3255 ± 0.0000	0.3255 ± 0.0000
#151675	**0.3890 ** ± ** 0.0067**	0.2221 ± 0.0807	0.1670 ± 0.0090	0.3371 ± 0.0132	0.3715 ± 0.0201	0.3210 ± 0.0041	0.3114 ± 0.0052	0.2006 ± 0.0000	0.3023 ± 0.0000	0.3024 ± 0.0000
#151676	**0.3367 ** ± ** 0.0198**	0.2572 ± 0.0082	0.0919 ± 0.0116	0.2914 ± 0.0114	0.2846 ± 0.0097	0.2675 ± 0.0109	0.2732 ± 0.0057	0.1745 ± 0.0000	0.2653 ± 0.0000	0.2654 ± 0.0000

Slices	Mean Squared Error (MSE)									
	SpaBiT	STAGE	HisToSGE	SpaViT	DIST	DeepSpaCE	ST-Net	NN	Linear	Cubic
#151507	**0.5406 ** ± ** 0.0195**	0.6254 ± 0.0040	0.5839 ± 0.0268	0.7124 ± 0.0094	0.8824 ± 0.0221	0.5750 ± 0.0038	0.5823 ± 0.0056	0.9003 ± 0.0000	0.7628 ± 0.0000	0.7629 ± 0.0000
#151508	0.5239 ± 0.0524	0.5406 ± 0.0105	0.5311 ± 0.0384	0.6518 ± 0.0152	0.8338 ± 0.0130	0.5283 ± 0.0047	**0.5198 ** ± ** 0.0063**	0.8071 ± 0.0000	0.6844 ± 0.0000	0.6544 ± 0.0000
#151509	**0.5751 ** ± ** 0.0492**	0.6665 ± 0.0122	0.6097 ± 0.0200	0.7548 ± 0.0088	0.9713 ± 0.0114	0.5930 ± 0.0093	0.5772 ± 0.0224	0.8876 ± 0.0000	0.7548 ± 0.0000	0.8548 ± 0.0000
#151510	**0.5500 ** ± ** 0.0367**	0.5969 ± 0.0052	0.5807 ± 0.0205	0.6554 ± 0.0294	0.7852 ± 0.0137	0.5741 ± 0.0060	0.5770 ± 0.0079	0.8690 ± 0.0000	0.7454 ± 0.0000	0.7454 ± 0.0000
#151669	**0.6237 ** ± ** 0.0589**	0.6976 ± 0.0072	0.6821 ± 0.0400	0.7135 ± 0.0091	0.8001 ± 0.0213	0.6642 ± 0.0143	0.6507 ± 0.0181	0.9780 ± 0.0000	0.7166 ± 0.0000	0.7166 ± 0.0000
#151670	**0.6211 ** ± ** 0.0789**	0.6503 ± 0.0074	0.6301 ± 0.0424	0.6501 ± 0.0147	0.7156 ± 0.0123	0.6173 ± 0.0229	0.6319 ± 0.0100	0.9563 ± 0.0000	0.6902 ± 0.0000	0.7003 ± 0.0000
#151671	**0.6270 ** ± ** 0.0301**	0.6973 ± 0.0068	0.8841 ± 0.0673	0.6844 ± 0.0241	0.8746 ± 0.0126	0.6311 ± 0.0139	0.6329 ± 0.0183	1.0014 ± 0.0000	0.6844 ± 0.0000	0.7245 ± 0.0000
#151672	**0.6255 ** ± ** 0.0189**	0.6827 ± 0.0048	0.6943 ± 0.0398	0.7132 ± 0.0152	0.8132 ± 0.0239	0.6596 ± 0.0376	0.6319 ± 0.0067	0.9913 ± 0.0000	0.7242 ± 0.0000	0.7362 ± 0.0000
#151673	**0.6498 ** ± ** 0.0446**	0.7429 ± 0.0057	0.7744 ± 0.0417	0.7341 ± 0.0163	0.8165 ± 0.0123	0.6713 ± 0.0140	0.6813 ± 0.0061	1.0455 ± 0.0000	0.7770 ± 0.0000	0.7579 ± 0.0000
#151674	**0.6318 ** ± ** 0.0114**	1.0027 ± 0.4845	0.7575 ± 0.0555	0.7426 ± 0.0328	0.8354 ± 0.0227	0.6597 ± 0.0085	0.6883 ± 0.0056	1.1059 ± 0.0000	0.7933 ± 0.0000	0.8011 ± 0.0000
#151675	**0.5945 ** ± ** 0.0427**	0.7484 ± 0.1026	0.7099 ± 0.0387	0.6841 ± 0.0106	0.7354 ± 0.0523	0.6082 ± 0.0066	0.6306 ± 0.0123	0.9661 ± 0.0000	0.6944 ± 0.0000	0.7044 ± 0.0000
#151676	**0.6217 ** ± ** 0.0244**	0.6498 ± 0.0231	0.6942 ± 0.0502	0.7321 ± 0.0149	0.7521 ± 0.0137	0.6361 ± 0.0076	0.6444 ± 0.0047	1.0035 ± 0.0000	0.7303 ± 0.0000	0.8304 ± 0.0000

Slices	Mean Absolute Error (MAE)									
	SpaBiT	STAGE	HisToSGE	SpaViT	DIST	DeepSpaCE	ST-Net	NN	Linear	Cubic
#151507	0.5773 ± 0.0017	0.6124 ± 0.0013	0.6091 ± 0.0035	0.6881 ± 0.0027	0.7166 ± 0.0065	**0.5457 ** ± ** 0.0028**	0.5612 ± 0.0029	0.6789 ± 0.0000	0.6901 ± 0.0000	0.6902 ± 0.0000
#151508	0.5422 ± 0.0112	0.5626 ± 0.0040	0.5642 ± 0.0055	0.6358 ± 0.0098	0.6477 ± 0.0054	**0.4899 ** ± ** 0.0019**	0.5976 ± 0.0062	0.6226 ± 0.0000	0.6578 ± 0.0000	0.6579 ± 0.0000
#151509	**0.5835 ** ± ** 0.0087**	0.5939 ± 0.0034	0.6245 ± 0.0051	0.6521 ± 0.0059	0.7153 ± 0.0054	0.6512 ± 0.0016	0.6523 ± 0.0036	0.6729 ± 0.0000	0.6954 ± 0.0000	0.6954 ± 0.0000
#151510	**0.5714 ** ± ** 0.0050**	0.5988 ± 0.0019	0.6063 ± 0.0092	0.6451 ± 0.0074	0.6617 ± 0.0037	0.6347 ± 0.0018	0.6425 ± 0.0032	0.6608 ± 0.0000	0.6891 ± 0.0000	0.6892 ± 0.0000
#151669	**0.6276 ** ± ** 0.0234**	0.6612 ± 0.0030	0.6688 ± 0.0056	0.7126 ± 0.0085	0.7435 ± 0.0069	0.6743 ± 0.0081	0.6681 ± 0.0053	0.7300 ± 0.0000	0.7361 ± 0.0000	0.7362 ± 0.0000
#151670	**0.6169 ** ± ** 0.0050**	0.6393 ± 0.0024	0.6525 ± 0.0042	0.6983 ± 0.0048	0.7961 ± 0.0095	0.6271 ± 0.0095	0.6391 ± 0.0059	0.7147 ± 0.0000	0.6952 ± 0.0000	0.7252 ± 0.0000
#151671	**0.6127 ** ± ** 0.0043**	0.6572 ± 0.0026	0.7279 ± 0.0959	0.6598 ± 0.0048	0.8598 ± 0.0064	0.6621 ± 0.0053	0.6515 ± 0.0083	0.7394 ± 0.0000	0.6758 ± 0.0000	0.7358 ± 0.0000
#151672	**0.6198 ** ± ** 0.0189**	0.6495 ± 0.0014	0.6712 ± 0.0102	0.6748 ± 0.0086	0.9074 ± 0.0076	0.6452 ± 0.0094	0.6352 ± 0.0022	0.7314 ± 0.0000	0.7343 ± 0.0000	0.7343 ± 0.0000
#151673	**0.6394 ** ± ** 0.0045**	0.6869 ± 0.0022	0.7199 ± 0.0054	0.6903 ± 0.0052	0.9280 ± 0.0084	0.6510 ± 0.0060	0.6594 ± 0.0023	0.7673 ± 0.0000	0.6992 ± 0.0000	0.7593 ± 0.0000
#151674	**0.6471 ** ± ** 0.0043**	0.7695 ± 0.1346	0.7150 ± 0.0168	0.7021 ± 0.0139	0.8126 ± 0.0093	0.6610 ± 0.0056	0.6714 ± 0.0038	0.80408 ± 0.0000	0.7789 ± 0.0000	0.7789 ± 0.0000
#151675	**0.6183 ** ± ** 0.0092**	0.6789 ± 0.0466	0.6701 ± 0.0062	0.6914 ± 0.0049	0.7070 ± 0.0035	0.6442 ± 0.0025	0.6218 ± 0.0035	0.7195 ± 0.0000	0.7284 ± 0.0000	0.7285 ± 0.0000
#151676	**0.6346 ** ± ** 0.0048**	0.6506 ± 0.0068	0.6878 ± 0.0113	0.7031 ± 0.0087	0.7659 ± 0.0095	0.6521 ± 0.0046	0.6637 ± 0.0042	0.7441 ± 0.0000	0.7464 ± 0.0000	0.7464 ± 0.0000

The best result is in bold font.

**Table 3:** Comparison of the performance of data generated by SpaBiT and baseline methods with the original data across three evaluation metrics on nine ST datasets.

Datasets	Pearson Correlation Coefficient (PCC)									
	SpaBiT	STAGE	HisToSGE	SpaViT	DIST	DeepSpaCE	ST-Net	NN	Linear	Cubic
MBSP	**0.5432 ** ± ** 0.0346**	0.1821 ± 0.0220	0.2571 ± 0.0084	0.4721 ± 0.0136	0.3318 ± 0.0231	0.4227 ± 0.0155	0.4570 ± 0.0128	0.5181 ± 0.0000	0.5268 ± 0.0000	0.5268 ± 0.0000
HCC	**0.6948 ** ± ** 0.0296**	0.1184 ± 0.0046	0.3899 ± 0.0300	0.5112 ± 0.0117	0.4183 ± 0.0226	0.4966 ± 0.0140	0.5326 ± 0.0121	0.4109 ± 0.0000	0.5106 ± 0.0000	0.5106 ± 0.0000
HIC	**0.4952 ** ± ** 0.0242**	0.4512 ± 0.0135	0.2947 ± 0.0179	0.4463 ± 0.0318	0.3967 ± 0.0169	0.4593 ± 0.0239	0.4778 ± 0.0127	0.4281 ± 0.0000	0.4374 ± 0.0000	0.4374 ± 0.0000
MP	**0.2373 ** ± ** 0.0479**	0.2243 ± 0.0063	0.2132 ± 0.1145	0.2639 ± 0.0000	0.2258 ± 0.0000	0.2166 ± 0.0049	0.2199 ± 0.0039	0.1162 ± 0.0000	0.1673 ± 0.0000	0.1673 ± 0.0000
HS	**0.4050 ** ± ** 0.0926**	0.2343 ± 0.0030	0.2279 ± 0.0243	0.3016 ± 0.0037	0.2963 ± 0.0249	0.2675 ± 0.0109	0.3238 ± 0.0108	0.2026 ± 0.0000	0.3562 ± 0.0000	0.3562 ± 0.0000
HBC_HD	**0.4132 ** ± ** 0.0418**	0.3034 ± 0.0275	0.4019 ± 0.0224	0.3332 ± 0.0117	0.3635 ± 0.0168	0.3354 ± 0.0024	0.3397 ± 0.0015	0.3008 ± 0.0000	0.3688 ± 0.0000	0.3594 ± 0.0000
MB_HD	**0.5512 ** ± ** 0.0111**	0.4989 ± 0.0234	0.4058 ± 0.0296	0.5168 ± 0.0237	0.4387 ± 0.0132	0.5345 ± 0.0020	0.4882 ± 0.0016	0.2876 ± 0.0000	0.3293 ± 0.0000	0.3171 ± 0.0000
HL1	**0.5114 ** ± ** 0.0194**	0.4603 ± 0.0500	0.4861 ± 0.0344	0.3321 ± 0.0332	0.3105 ± 0.0158	0.5087 ± 0.0011	0.4938 ± 0.0015	0.3462 ± 0.0000	0.3197 ± 0.0000	0.3557 ± 0.0000
HL2	**0.2914 ** ± ** 0.0154**	0.2153 ± 0.0378	0.2020 ± 0.0150	0.2185 ± 0.0221	0.2237 ± 0.0147	0.2326 ± 0.0012	0.2308 ± 0.0016	0.1845 ± 0.0000	0.1701 ± 0.0000	0.1887 ± 0.0000

Datasets	Mean Squared Error (MSE)									
	SpaBiT	STAGE	HisToSGE	SpaViT	DIST	DeepSpaCE	ST-Net	NN	Linear	Cubic
MBSP	**0.8365 ** ± ** 0.0767**	1.2853 ± 0.0320	1.3901 ± 0.0346	1.0081 ± 0.0147	1.0132 ± 0.0268	1.1453 ± 0.0304	1.1294 ± 0.0156	1.2282 ± 0.0000	0.8953 ± 0.0000	0.9962 ± 0.0000
HCC	**0.7829 ** ± ** 0.1272**	1.3841 ± 0.0366	1.2370 ± 0.0821	1.1425 ± 0.0127	1.2331 ± 0.0317	1.0615 ± 0.0404	1.0004 ± 0.0329	1.0238 ± 0.0000	0.8586 ± 0.0000	0.9513 ± 0.0000
HIC	1.0680 ± 0.0291	1.2825 ± 0.0503	1.2127 ± 0.0227	1.1873 ± 0.0225	1.1375 ± 0.0169	1.1144 ± 0.0267	1.1970 ± 0.0121	1.1546 ± 0.0000	0.9381 ± 0.0000	**0.8962 ** ± ** 0.0000**
MP	0.3651 ± 0.0440	0.3867 ± 0.0023	0.4599 ± 0.0093	0.4012 ± 0.0131	0.3786 ± 0.0179	**0.3411 ** ± ** 0.0064**	0.3470 ± 0.0032	0.6153 ± 0.0000	0.3982 ± 0.0000	0.3982 ± 0.0000
HS	**0.3280 ** ± ** 0.0258**	0.3825 ± 0.0047	0.3480 ± 0.0889	0.3365 ± 0.0118	0.4218 ± 0.0114	0.4588 ± 0.0001	0.4691 ± 0.0082	0.4575 ± 0.0000	0.5639 ± 0.0000	0.4639 ± 0.0000
HBCHD	**0.6467 ** ± ** 0.0344**	0.9978 ± 0.0514	0.6938 ± 0.0302	0.6223 ± 0.0163	0.6531 ± 0.0136	0.7718 ± 0.0028	0.6539 ± 0.0037	0.9342 ± 0.0000	0.6309 ± 0.0000	1.2823 ± 0.0000
MBHD	**0.5966 ** ± ** 0.0362**	0.7145 ± 0.0595	1.0364 ± 0.0567	0.9006 ± 0.0172	0.9163 ± 0.0169	0.6553 ± 0.0065	0.6347 ± 0.0035	1.1237 ± 0.0000	0.9670 ± 0.0000	1.6409 ± 0.0000
HL1	**0.4857 ** ± ** 0.0047**	0.5433 ± 0.0698	0.5695 ± 0.0390	0.8867 ± 0.0173	0.8549 ± 0.0128	0.5336 ± 0.0012	0.5280 ± 0.0017	0.7912 ± 0.0000	0.8902 ± 0.0000	1.5455 ± 0.0000
HL2	**0.3593 ** ± ** 0.0231**	0.3764 ± 0.0125	0.3664 ± 0.0070	0.5631 ± 0.0127	0.4658 ± 0.0307	0.4731 ± 0.0019	0.4693 ± 0.0022	0.3678 ± 0.0000	0.5514 ± 0.0000	1.0959 ± 0.0000

Datasets	Mean Absolute Error (MAE)									
	SpaBiT	STAGE	HisToSGE	SpaViT	DIST	DeepSpaCE	ST-Net	NN	Linear	Cubic
MBSP	**0.7056 ** ± ** 0.0331**	1.1278 ± 0.0690	0.9277 ± 0.0126	0.8002 ± 0.0126	0.8102 ± 0.0226	0.8302 ± 0.0101	0.8241 ± 0.0057	1.2282 ± 0.0000	0.7985 ± 0.0000	0.7964 ± 0.0000
HCC	**0.6599 ** ± ** 0.0360**	1.2185 ± 0.6021	0.8793 ± 0.0328	0.8191 ± 0.0117	0.8265 ± 0.0198	0.8070 ± 0.0172	0.7809 ± 0.0062	1.0238 ± 0.0000	0.8347 ± 0.0000	0.9347 ± 0.0000
HIC	**0.8106 ** ± ** 0.0409**	1.1203 ± 0.3410	0.8583 ± 0.0087	0.8812 ± 0.0163	0.8759 ± 0.0177	0.8712 ± 0.0118	1.1546 ± 0.0213	1.1546 ± 0.0000	0.8627 ± 0.0000	0.9627 ± 0.0000
MP	**0.2977 ** ± ** 0.0048**	0.3303 ± 0.0030	0.3179 ± 0.0099	0.3315 ± 0.0152	0.3514 ± 0.0164	0.3564 ± 0.0018	0.3601 ± 0.0004	0.6153 ± 0.0000	0.3684 ± 0.0000	0.3681 ± 0.0000
HS	**0.2921 ** ± ** 0.0191**	0.2958 ± 0.0041	0.2684 ± 0.0095	0.2997 ± 0.0194	0.3117 ± 0.0173	0.3775 ± 0.0011	0.3377 ± 0.0036	0.4575 ± 0.0000	0.3047 ± 0.0000	0.3244 ± 0.0000
HBCHD	**0.4745 ** ± ** 0.0106**	0.6114 ± 0.0153	0.5165 ± 0.0153	0.4974 ± 0.0193	0.5621 ± 0.0159	0.5468 ± 0.0019	0.5472 ± 0.0023	0.5529 ± 0.0000	0.5918 ± 0.0000	1.1927 ± 0.0000
MBHD	**0.5233 ** ± ** 0.0088**	0.5866 ± 0.0163	0.6098 ± 0.0131	0.6257 ± 0.0186	0.5946 ± 0.0138	0.5293 ± 0.0030	0.5302 ± 0.0014	0.6302 ± 0.0000	0.5736 ± 0.0000	1.5110 ± 0.0000
HL1	**0.4197 ** ± ** 0.0030**	0.4543 ± 0.0243	0.4875 ± 0.0113	0.6011 ± 0.0176	0.6123 ± 0.0185	0.4506 ± 0.0008	0.4526 ± 0.0010	0.5684 ± 0.0000	0.5597 ± 0.0000	1.0404 ± 0.0000
HL2	**0.3230 ** ± ** 0.0071**	0.3413 ± 0.0043	0.3364 ± 0.0035	0.4652 ± 0.0163	0.4795 ± 0.0176	0.3753 ± 0.0010	0.3774 ± 0.0019	0.4606 ± 0.0000	0.4048 ± 0.0000	1.2360 ± 0.0000

The best result is in bold font.

To intuitively compare the performance of different methods in gene expression reconstruction, we selected multiple marker genes and visualized their gene expression profiles on the MBHD slice. We plotted their spatial expression patterns in both the original data and the data generated by each method to assess the degree of pattern restoration. Visualization results for additional datasets are provided in Supplementary Materials S6, available as supplementary data at *Bioinformatics* online.

**Figure 2:**
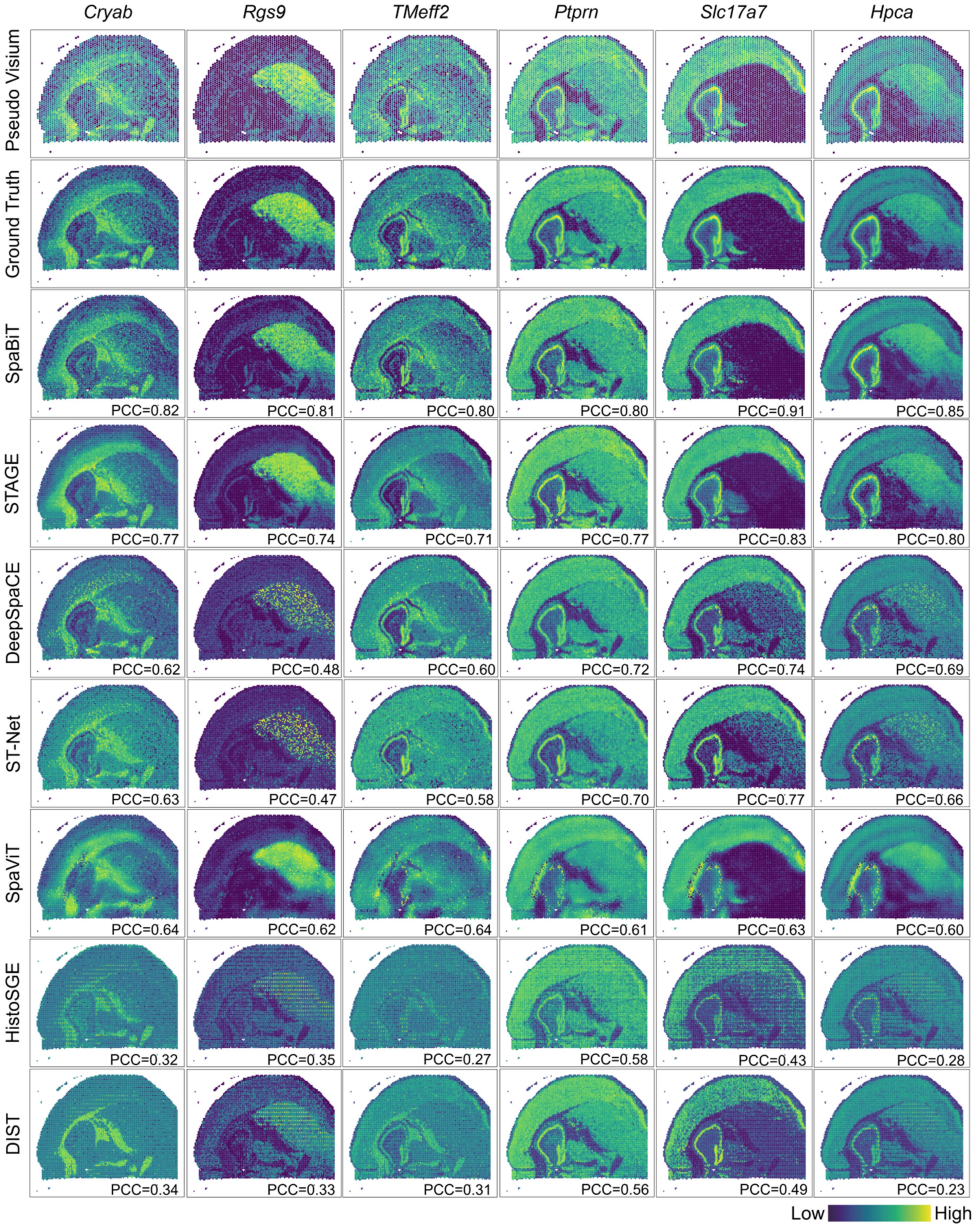
Spatial visualization of genes with different spatial patterns (*Cryab*, *Rgs*9, *TMeff*2, *Ptprn*, *Slc*17*a*7, *Hpca*) for the pseudo Visium, ground truth, and predicted high-resolution gene expression profiles by SpaBiT, STAGE, DeepSpaCE, ST-Net, HisToSGE, SpaViT, and DIST in MBHD data.

SpaBiT demonstrates significantly better preservation of spatial gene expression profiles compared to baseline methods, effectively recovering both global expression trends and local spatial variations. As shown in the visualization results (Fig. 2), the gene expression patterns reconstructed by SpaBiT align more closely with the original data with higher PCC values. These results collectively show that SpaBiT effectively leverages histological image features and spatial context to enhance the accuracy and reliability of gene expression reconstruction.

### 3.4 SpaBiT can better preserve spatial structures

**Figure 3:**
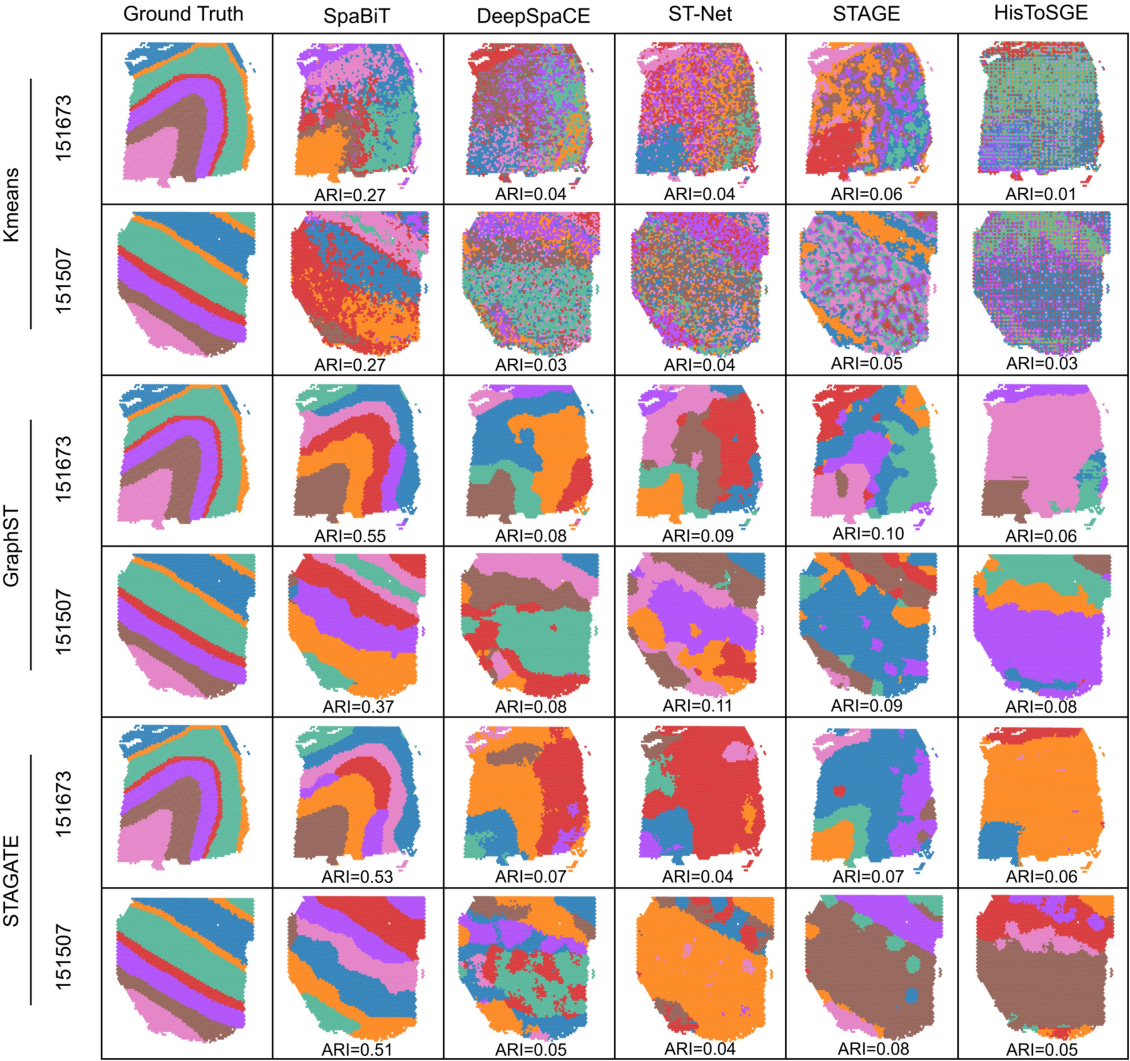
Spatial domain identification on the 10x Genomics DLPFC dataset, comparing the proposed method SpaBiT with baseline approaches DeepSpaCE, ST-Net, STAGE, and HisToSGE. Columns show the ground-truth annotations and the clustering results of each method, while rows correspond to different clustering methods (K-means, GraphST, and STAGATE) applied to two representative slices (151673 and 151507). The ARI with respect to the histological annotations is reported under each panel.

To assess the ability of the proposed method to preserve spatial structures, we performed clustering analysis on two representative slices (151673 and 151507) from the 10× Genomics DLPFC dataset (Fig. 3). The histologically annotated cortical layers were used as ground truth, and the Adjusted Rand Index (ARI) was adopted to quantify clustering performance. We applied three commonly used clustering strategies, including K-means, GraphST (Long *et al.* 2023) and STAGATE (Dong and Zhang 2022), to the low-dimensional embeddings produced by our model and four baseline methods (DeepSpaCE, ST-Net, STAGE, and HisToSGE).

For K-means clustering, our method achieved ARI scores of 0.27 on both slices 151673 and 151507, clearly outperforming all competitors, whose ARIs ranged from 0.01 to 0.06. When using GraphST, our embeddings yielded ARIs of 0.55 and 0.37 on the two slices, whereas DeepSpaCE, ST-Net, STAGE, and HisToSGE only reached 0.08–0.11. A similar trend was observed with STAGATE: our method obtained ARIs of 0.53 and 0.51, which were consistently higher than the baselines (0.04–0.08). These slice-wise results demonstrate that, under all three clustering backbones, the representations learned by our method are substantially more informative for spatial domain recognition. For both slices, clusters derived from our method delineate layered cortical structures that closely match the ground-truth annotations, with clear and smooth boundaries between adjacent domains. These results indicate that our method can more accurately and robustly recover biologically meaningful spatial domains from STs data.

### 3.5 Identify tumor regions using SpaBiT

**Figure 4:**
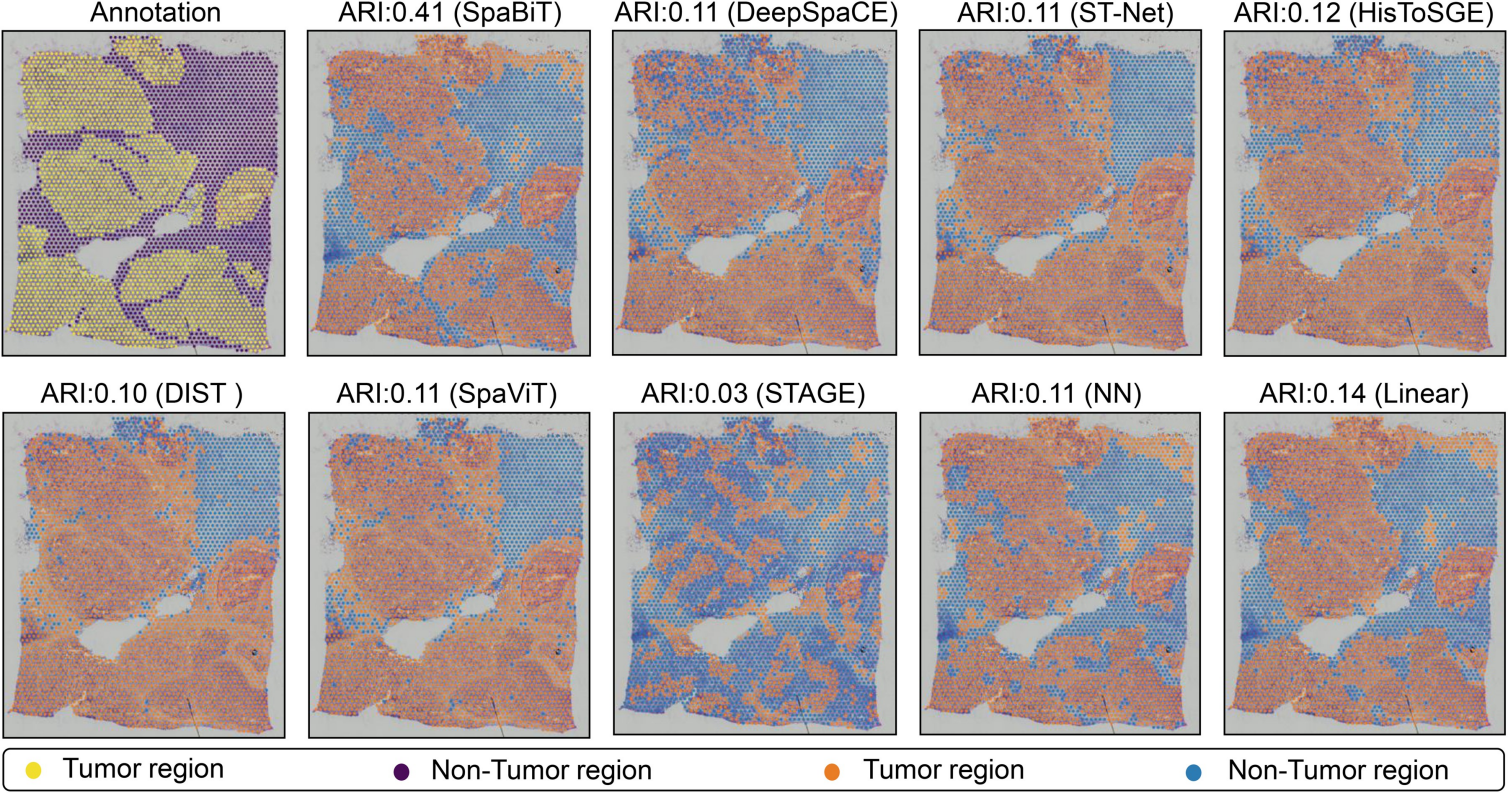
Clustering analysis of predicted gene expression profiles on the BC slice. Spots were grouped into cancerous and normal tissue regions based on pathologist annotations.

To evaluate whether predicted gene expression profiles can support accurate identification of tumor region, we conducted binary clustering on the BC slice. For each method, K-means clustering with *K* = 2 was applied to the predicted gene expression profiles, and the two clusters were interpreted as tumor and non-tumor regions. Pathologist annotations were used as ground truth, and the ARI was calculated to assess the consistency between clustering results and manual labels (Fig. 4). SpaBiT achieved an ARI of 0.41, which is clearly higher than all baseline approaches, whose ARIs are approximately 0.10–0.14. The visualization shows that SpaBiT produces tumor maps that closely follow the annotated cancerous and normal tissue regions, indicating that our method preserves more discriminative information for downstream tumor region identification.

### 3.6 Explore biological functions of cancer data using SpaBiT

**Figure 5:**
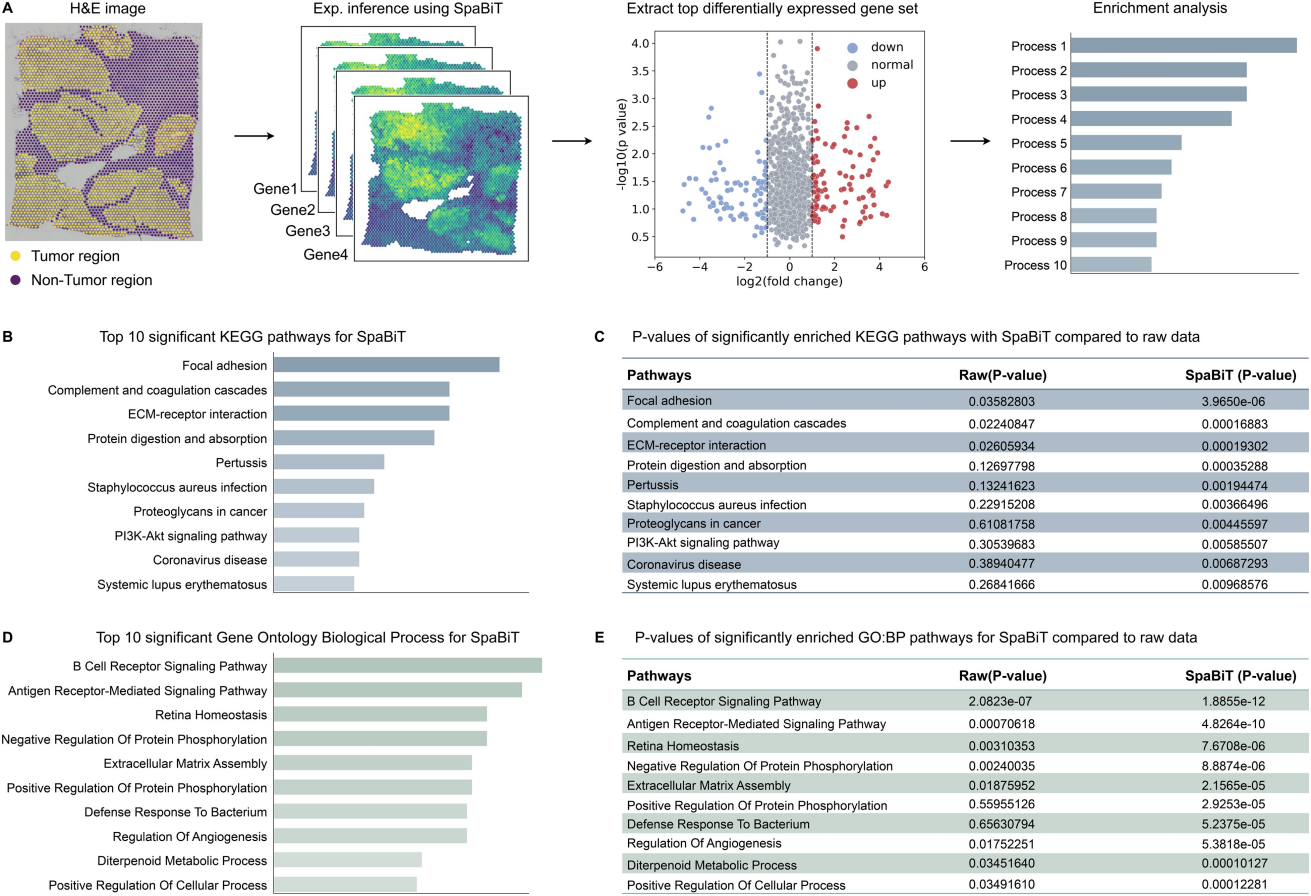
KEGG and GO: BP pathway enrichment analysis of DEGs between tumor and non-tumor regions based on gene expression predicted by SpaBiT on the BC slice. (a) Workflow for identifying DEGs and performing enrichment analysis using SpaBiT-reconstructed gene expression. (b) and (c) show the *P*-value of significantly enriched KEGG pathways obtained from SpaBiT-reconstructed expression compared with those from the raw spatial transcriptomics data. (d) and (e) display the *P*-value of significantly enriched GO: BP pathways identified using SpaBiT-reconstructed expression versus the raw data.

To evaluate whether SpaBiT-inferred gene expression profiles can reveal meaningful biological signals, we performed pathway enrichment analysis on the BC slice (Fig. 5). We carried out differential expression analysis by treating the tumor region as the case group and the non-tumor region as the reference group, and applying a Wilcoxon rank-sum test to each gene. For every gene, we computed the fold change on a log-transformed scale, together with the raw *P*-value and the Benjamini–Hochberg adjusted *P-*value (Chen and Sarkar 2020). Genes with an adjusted *P-*value below 0.05 and an absolute log-scaled fold change larger than one were defined as significantly differentially expressed genes (DEGs). According to the sign of the fold change, these DEGs were further labeled as upregulated or downregulated. The resulting set of significant DEGs was then used as the input gene list for subsequent KEGG (Kanehisa *et al.* 2023) and GO enrichment analyses (Kolberg *et al.* 2023).

Using the DEGs between tumor and non-tumor regions, we compared pathway enrichment based on SpaBiT-reconstructed expression profiles and on the original STs data. Overall, SpaBiT led to consistently smaller enrichment *P-*value across the top pathways, indicating that the reconstructed expression profiles provide a clearer and more concentrated cancer-related signal. In the KEGG analysis, pathways such as focal adhesion, ECM–receptor interaction, proteoglycans in cancer, complement and coagulation cascades, and PI3K–Akt signaling showed greater enrichment when using SpaBiT-reconstructed expression than when using raw data. Focal adhesion and ECM-related signaling are known to regulate cell adhesion, migration and invasion, and are key drivers of BC progression through focal adhesion kinase and other adhesion-related molecules (Rigiracciolo *et al.* 2021). The extracellular matrix and its receptors also form a critical component of the tumor microenvironment and actively shape metastasis and therapeutic response (Closset *et al.* 2023). The PI3K–Akt axis is one of the most frequently activated signaling pathways in BC and promotes tumor cell growth, survival, and angiogenesis, making it a major therapeutic target (Zhang *et al.* 2024a). In addition, complement and coagulation cascades, as well as proteoglycans in cancer, became more significant in the SpaBiT-based enrichment. These pathways are increasingly recognized as regulators of the tumor microenvironment, modulating thrombo-inflammatory responses, immune cell infiltration, and metastatic behavior (Bauer *et al.* 2022). Consistently, GO Biological Process enrichment based on SpaBiT highlighted terms related to extracellular matrix organization, regulation of angiogenesis, and immune-related processes such as antigen receptor–mediated signaling and B-cell–mediated responses. Extracellular matrix remodeling and angiogenesis are essential for breast tumor growth and metastasis, and have clear prognostic and therapeutic implications (Libby *et al.* 2024). Immune-related processes, including B cell receptor signaling and antigen presentation, are also increasingly appreciated as key components of the immune microenvironment of BC (Ye and Lee 2022). These results indicate that SpaBiT not only preserves but also amplifies biologically meaningful signals linked to BC progression.

### 3.7 Explore insights by high-resolution gene expression

To systematically evaluate spatial gene patterns, we use Sepal, which assigns each gene a score based on the strength of its spatial organization (Andersson and Lundeberg 2021). Sepal was applied in parallel to the raw HER2ST data and the SpaBiT-enhanced expression profiles of section E1.

**Figure 6:**
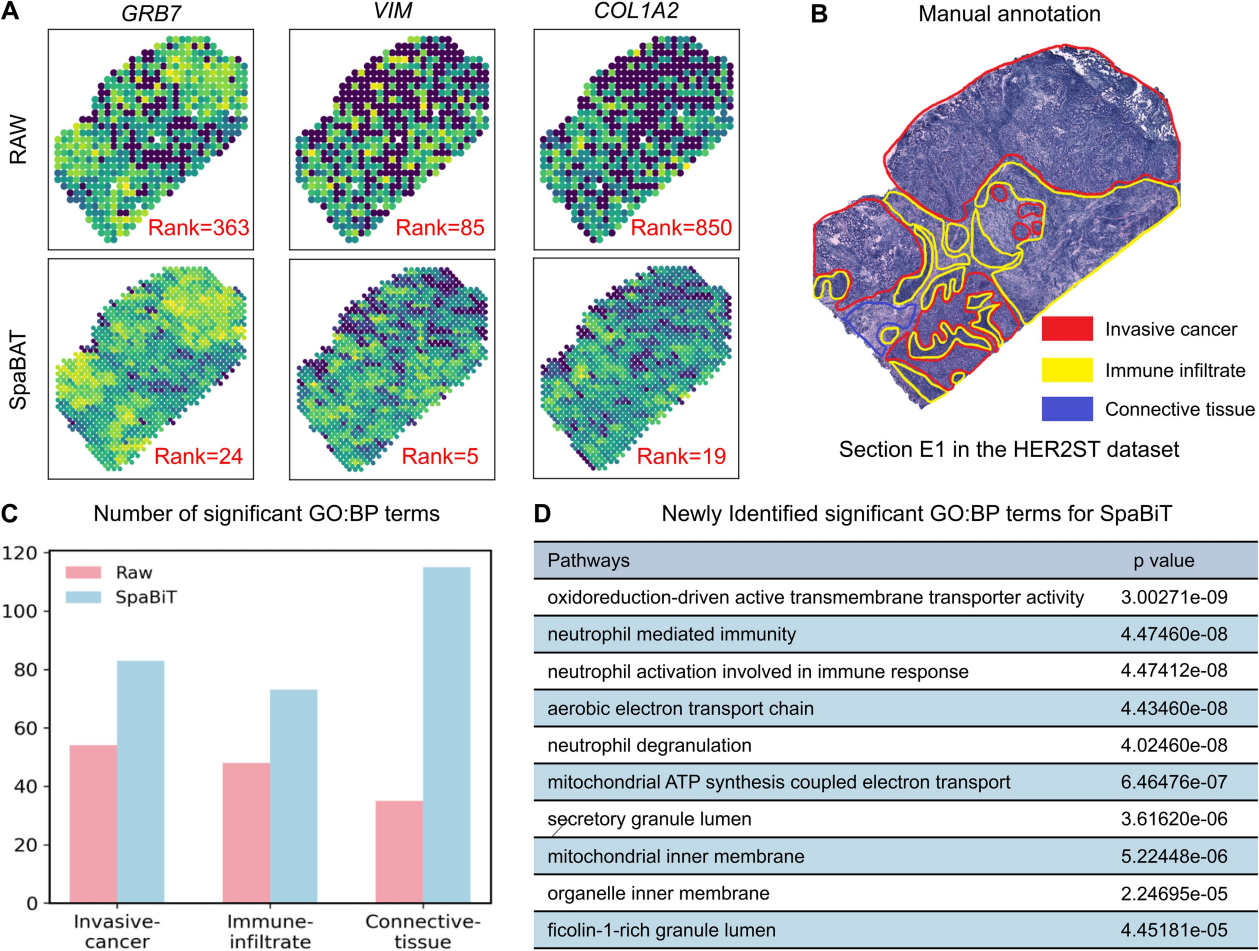
SpaBiT reveals new insights in HER2ST data. (a) Examples of disease-associated genes that are ranked higher in the SpaBiT-enhanced data but much lower in the raw data. (b) Manual annotations for section E1 of the HER2ST dataset, including invasive cancer, immune infiltrate and connective tissue regions. (c) Number of significant GO: BP terms (*P*-value 0.05) identified in each tissue family of section E1 for raw data and SpaBiT-enhanced data. (d) Newly identified significant GO: BP terms using SpaBiT in section E1 of the HER2ST dataset.

As shown in Fig. 6a, SpaBiT markedly improved the spatial ranking of several cancer-related genes, including *GRB*7, *VIM*, and *COL*1*A*2. *GRB*7 is frequently overexpressed in HER2-positive BC and is associated with poor prognosis as well as enhanced proliferation, migration and invasion of tumor cells (Wang *et al.* 2024). Vimentin (*VIM*) is a canonical marker of epithelial-to-mesenchymal transition and plays a central role in cancer progression and metastasis (Tabatabaee *et al.* 2024).*COL*1*A*2 encodes a chain of type I collagen, a major stromal component in the tumor microenvironment whose abundance and organization are tightly linked to BC progression and clinical outcome (Heydari *et al.* 2025). In the raw expression profiles, these genes exhibit blurred and fragmented spatial distributions, resulting in low Sepal scores. After SpaBiT enhancement, they display coherent patterns highly consistent with histological structures, leading to a significant jump in their rankings. This indicates that SpaBiT enhances the spatial organization of key cancer-related genes and makes their functional roles in the tissue context easier to interpret.

Section E1 was manually annotated as invasive-cancer, immune-infiltrate, and connective-tissue regions (Fig. 6b). For each region, we selected the top 150 spatially patterned genes based on Sepal scores from both raw and SpaBiT-enhanced profiles to perform GO: Biological Process (GO: BP) enrichment analysis.

As shown in Fig. 6c, SpaBiT yields a larger number of significant GO terms across all regions. Further global analysis (Fig. 6d) identified key biological processes tightly linked to tumor biology. For instance, terms related to neutrophil-mediated immunity and degranulation reflect the dual roles of neutrophils in the tumor microenvironment (Zheng *et al.* 2022). Additionally, processes such as mitochondrial ATP (Adenosine Triphosphate) synthesis coupled electron transport highlight the importance of oxidative phosphorylation in supporting cancer cell survival and therapy resistance (Uslu *et al.* 2024). In addition, pathways associated with the secretory granule lumen are consistent with the emerging role of the tumor and immune cell secretome in remodeling the tumor microenvironment and modulating antitumor immunity (Rawat *et al.* 2021).

Together, these results demonstrate that SpaBiT-enhanced high-resolution expression not only improves the spatial detectability of known cancer genes but also strengthens the enrichment of biologically relevant pathways, providing new insights into tumor–immune interactions and potential therapeutic targets in HER2-positive BC.

### 3.8 Ablation studies

To better understand the role of each module in SpaBiT, we performed three groups of ablation experiments on 10 STs datasets.

The first ablation investigates different attention strategies for multimodal fusion between histology and neighborhood features (Table 4). The full SpaBiT model, equipped with bidirectional CA to jointly integrate UNI-based image embeddings and GAT-derived neighborhood features, achieves the best overall performance. Removing the image attention branch leads to a clear drop in PCC and an increase in reconstruction error, indicating that histological context—captured by the UNI model—provides complementary morphological information that cannot be fully substituted by local expression structure, which is critical for resolving fine-grained spatial heterogeneities in complex tissues. Removing the neighbor attention branch also degrades performance, showing that explicitly modeling spatial neighborhoods is important even when strong image features are available. When both branches are removed, the model degenerates into a simple mapping without cross-modal interaction, and the predictive accuracy is further reduced, highlighting the necessity of bidirectional fusion between the two modalities. To further evaluate alternative fusion strategies, we compared our bidirectional CA framework against standard non-attention baselines, including feature concatenation and weighted element-wise fusion. These extended evaluations are detailed in the Supplementary Materials S7, available as supplementary data at *Bioinformatics* online.

**Table 4:** Ablation study of SpaBiT on ten spatial transcriptomics datasets, where different attention strategies are used for multimodal feature fusion.

**PCC** ↑	DLPFC	MBSP	HCC	HIC	MP	HS	HBCHD	MBHD	HL1	HL2
SpaBiT	**0.3317 ** ± ** 0.0567**	**0.6268 ** ± ** 0.0377**	**0.6859 ** ± ** 0.0258**	**0.5420 ** ± ** 0.0128**	**0.2768 ** ± ** 0.0664**	**0.3682 ** ± ** 0.0131**	**0.5099 ** ± ** 0.0156**	**0.6661 ** ± ** 0.0161**	**0.5574 ** ± ** 0.0258**	**0.3514 ** ± ** 0.0112**
(w/o) img	0.1472 ± 0.0368	0.3097 ± 0.0920	0.2954 ± 0.0298	0.2255 ± 0.0806	0.1721 ± 0.1427	0.1268 ± 0.0213	0.3363 ± 0.0077	0.4487 ± 0.0268	0.5109 ± 0.0208	0.3372 ± 0.0167
(w/o) nbr	0.1623 ± 0.0386	0.2144 ± 0.0221	0.2974 ± 0.0124	0.3192 ± 0.0124	0.2402 ± 0.1738	0.2671 ± 0.0165	0.4532 ± 0.0088	0.4541 ± 0.1631	0.4841 ± 0.0079	0.3222 ± 0.0086
(w/o) img&nbr	0.1397 ± 0.0245	0.2571 ± 0.0274	0.2671 ± 0.1022	0.0081 ± 0.0133	0.0891 ± 0.1204	0.0956 ± 0.0169	0.0019 ± 0.0071	0.1070 ± 0.1825	0.0040 ± 0.0135	0.0025 ± 0.0079
**MSE** ↓	DLPFC	MBSP	HCC	HIC	MP	HS	HBCHD	MBHD	HL1	HL2
SpaBiT	**0.5987 ** ± ** 0.0432**	**0.8365 ** ± ** 0.0767**	**0.7829 ** ± ** 0.1272**	**1.0680 ** ± ** 0.0291**	**0.3651 ** ± ** 0.0440**	**0.3403 ** ± ** 0.0818**	**0.6467 ** ± ** 0.0344**	**0.5966 ** ± ** 0.0362**	**0.4857 ** ± ** 0.0047**	**0.3593 ** ± ** 0.0231**
(w/o) img	0.6510 ± 0.0512	0.9067 ± 0.0485	0.9913 ± 0.0267	1.1866 ± 0.0258	0.4586 ± 0.0310	0.3758 ± 0.0845	0.6914 ± 0.0150	0.9267 ± 0.0398	0.5315 ± 0.0334	0.4245 ± 0.0125
(w/o) nbr	0.6827 ± 0.0678	0.9386 ± 0.0569	0.9532 ± 0.0458	1.1740 ± 0.0264	0.4420 ± 0.0354	0.3752 ± 0.0310	1.0065 ± 0.0069	0.8369 ± 0.1638	0.9115 ± 0.0450	0.4426 ± 0.0230
(w/o) img&nbr	0.6486 ± 0.0463	0.9222 ± 0.0162	0.9928 ± 0.0271	1.2124 ± 0.0311	0.3845 ± 0.0345	0.3999 ± 0.0659	0.7486 ± 0.0143	0.9613 ± 0.1840	0.5300 ± 0.0061	0.4149 ± 0.0076
**MAE** ↓	DLPFC	MBSP	HCC	HIC	MP	HS	HBCHD	MBHD	HL1	HL2
SpaBiT	**0.6076 ** ± ** 0.0105**	**0.7056 ** ± ** 0.0331**	**0.6747 ** ± ** 0.0384**	**0.7983 ** ± ** 0.0106**	**0.2977 ** ± ** 0.0048**	**0.2667 ** ± ** 0.0088**	**0.4745 ** ± ** 0.0106**	**0.4421 ** ± ** 0.0131**	**0.4197 ** ± ** 0.0030**	**0.3230 ** ± ** 0.0071**
(w/o) img	0.6551 ± 0.0227	0.8033 ± 0.0315	0.7980 ± 0.0209	0.8018 ± 0.0169	0.3301 ± 0.0125	0.3673 ± 0.0059	0.4989 ± 0.0031	0.5912 ± 0.0097	0.5121 ± 0.0086	0.3691 ± 0.0035
(w/o) nbr	0.6968 ± 0.0673	0.8250 ± 0.0332	0.7385 ± 0.0302	0.8982 ± 0.0169	0.3497 ± 0.0168	0.3021 ± 0.0180	0.6464 ± 0.0044	0.6839 ± 0.0538	0.6081 ± 0.0084	0.3486 ± 0.0079
(w/o) img&nbr	0.6548 ± 0.0326	0.8124 ± 0.0094	0.7947 ± 0.0180	0.8139 ± 0.0196	0.3042 ± 0.0248	0.3635 ± 0.0068	0.4944 ± 0.0068	0.5879 ± 0.0699	0.5008 ± 0.0036	0.3923 ± 0.0021

The best result in each column is in bold font.

The second ablation focuses on the choice of the image encoder by replacing UNI with Conch, DenseNet, VGG16, and ResNet while keeping the rest of SpaBiT unchanged (Table 5). Using UNI as the backbone consistently yields a higher PCC and lower error than the alternative encoders, with especially pronounced advantages in more challenging datasets. This suggests that large-scale pathology foundation models provide richer and more robust visual representations for spatial gene expression prediction than conventional CNN-based backbones or smaller-scale pre-training and that SpaBiT is able to effectively exploit these high-quality image features.

**Table 5:** Ablation study on the image encoder of SpaBiT over ten spatial transcriptomics datasets, where different backbone models (UNI, Conch, DenseNet, VGG16, ResNet) are used for histology feature extraction.

**PCC** ↑	DLPFC	MBSP	HCC	HIC	MP	HS	HBCHD	MBHD	HL1	HL2
(w)UNI	**0.3317 ** ± ** 0.0567**	**0.5432 ** ± ** 0.0346**	**0.6948 ** ± ** 0.0296**	**0.4952 ** ± ** 0.0242**	**0.2373 ** ± ** 0.0479**	0.4050 ± 0.0926	**0.4132 ** ± ** 0.0418**	**0.5512 ** ± ** 0.0111**	**0.5114 ** ± ** 0.0194**	**0.2914 ** ± ** 0.0154**
(w)Conch	0.2762 ± 0.0989	0.4900 ± 0.1233	0.5567 ± 0.2214	0.4721 ± 0.0381	0.1963 ± 0.0345	0.3461 ± 0.0911	0.3581 ± 0.0455	0.5262 ± 0.0228	0.4880 ± 0.0332	0.2808 ± 0.0279
(w)DenseNet	0.2839 ± 0.1166	0.5047 ± 0.1023	0.5895 ± 0.1551	0.4687 ± 0.2190	0.2035 ± 0.0237	0.3014 ± 0.0236	0.3864 ± 0.0127	0.5105 ± 0.0413	0.4100 ± 0.1576	0.2527 ± 0.0300
(w)VGG16	0.2685±0.0899	0.2870±0.2285	0.5123±0.1291	0.3837±0.0981	0.1896±0.0152	**0.4307** ± **0.1044**	0.3606±0.0109	0.5331±0.0228	0.4734±0.0224	0.2579±0.0402
(w)ResNet	0.2773 ± 0.1037	0.4921 ± 0.2281	0.3039 ± 0.3194	0.2805 ± 0.2321	0.2312 ± 0.0382	0.3527 ± 0.1047	0.2383 ± 0.2153	0.4654 ± 0.0431	0.5015 ± 0.0543	0.2902 ± 0.0809
**MSE** ↓	DLPFC	MBSP	HCC	HIC	MP	HS	HBCHD	MBHD	HL1	HL2
(w)UNI	**0.5987 ** ± ** 0.0432**	**0.8365 ** ± ** 0.0767**	**0.7829 ** ± ** 0.1272**	1.0680 ± 0.0291	**0.3651 ** ± ** 0.0440**	**0.3280 ** ± ** 0.0258**	**0.6467 ** ± ** 0.0344**	**0.5966 ** ± ** 0.0362**	**0.4857 ** ± ** 0.0047**	**0.3593 ** ± ** 0.0231**
(w)Conch	0.6527 ± 0.0530	1.2064 ± 0.2954	0.9805 ± 0.2144	**0.8942 ** ± ** 0.0515**	0.4689 ± 0.0376	0.3576 ± 0.0281	0.9122 ± 0.0782	0.7689 ± 0.0789	0.5917 ± 0.0412	0.4247 ± 0.0440
(w)DenseNet	0.6948 ± 0.1711	1.0900 ± 0.1640	0.9723 ± 0.3762	1.1136 ± 0.2267	0.4662 ± 0.0426	0.3347 ± 0.0615	0.8954 ± 0.0387	0.8565 ± 0.0332	0.6748 ± 0.1001	0.4308 ± 0.0407
(w)VGG16	0.6484±0.0634	1.4580±0.3352	2.8675±1.8317	1.2730±0.1106	0.4816±0.0232	0.3533±0.0632	0.9386±0.0356	0.8269±0.0724	0.6033±0.0304	0.4354±0.0403
(w)ResNet	0.6551 ± 0.0862	1.0235 ± 0.1962	1.2600 ± 0.3752	1.2449 ± 0.2487	0.4261 ± 0.0445	0.3520 ± 0.0651	0.9285 ± 0.0758	0.7660 ± 0.0628	0.5873 ± 0.0632	0.4030 ± 0.0584
**MAE** ↓	DLPFC	MBSP	HCC	HIC	MP	HS	HBCHD	MBHD	HL1	HL2
(w)UNI	**0.6076 ** ± ** 0.0105**	**0.7056 ** ± ** 0.0331**	**0.6599 ** ± ** 0.0360**	0.8106 ± 0.0409	**0.2977 ** ± ** 0.0048**	**0.2921 ** ± ** 0.0191**	**0.4745 ** ± ** 0.0106**	**0.5233 ** ± ** 0.0088**	**0.4197 ** ± ** 0.0030**	**0.3230 ** ± ** 0.0071**
(w)Conch	0.6238 ± 0.0197	0.8434 ± 0.1150	0.7505 ± 0.1074	**0.7103 ** ± ** 0.0170**	0.3537 ± 0.0075	0.3037 ± 0.0233	0.5933 ± 0.0290	0.5674 ± 0.0258	0.4639 ± 0.0121	0.3473 ± 0.0152
(w)DenseNet	0.6382 ± 0.0462	0.7879 ± 0.0581	0.7502 ± 0.1383	0.8659 ± 0.0912	0.3439 ± 0.0083	0.3100 ± 0.0225	0.5857 ± 0.0086	0.5339 ± 0.0102	0.4988 ± 0.0498	0.3535 ± 0.0154
(w)VGG16	0.6296±0.0227	0.9288±0.1149	1.2390±0.4649	0.8698±0.0467	0.3521±0.0055	0.2990±0.0253	0.6015±0.0070	0.5265±0.0200	0.4697±0.0101	0.3539±0.0114
(w)ResNet	0.6290 ± 0.0235	0.7766 ± 0.0872	0.8570 ± 0.1417	0.8554 ± 0.0961	0.3510 ± 0.0101	0.2970 ± 0.0277	0.6021 ± 0.0313	0.5600 ± 0.0188	0.4644 ± 0.0222	0.3420 ± 0.0213

The best result in each column is in bold font.

The third ablation examines neighborhood feature extraction strategies by comparing GAT with GCN, a generic variant of GNN, and a simple mean-filling method (Table 6). Encoding the spatial graph with GAT leads to the most favorable trade-off between correlation and error, reflecting the benefit of adaptive attention weights when aggregating neighbor information. The GCN and generic GNN variants capture part of the spatial structure but generally underperform GAT, while the mean-filling approach, which aggregates neighbor expressions without any learnable graph parameters, shows the weakest performance. These results indicate that attention-based neighborhood modeling is an important component of SpaBiT and plays a key role in stabilizing prediction quality across heterogeneous tissues. To evaluate parameter sensitivity, the impact of neighbor count *K* was investigated across different slices, with detailed ablation results and rationale provided in Supplementary Materials S8, available as supplementary data at *Bioinformatics* online.

**Table 6:** Ablation study on neighborhood feature extraction in SpaBiT across ten spatial transcriptomics datasets, comparing different graph neural network encoders (GAT, GCN, generic GNN variants) and a mean-filling baseline.

**PCC** ↑	DLPFC	MBSP	HCC	HIC	MP	HS	HBCHD	MBHD	HL1	HL2
(w) GAT	**0.3317 ** ± ** 0.0567**	**0.5432 ** ± ** 0.0346**	**0.6948 ** ± ** 0.0296**	**0.4952 ** ± ** 0.0242**	**0.2373 ** ± ** 0.0479**	**0.4050 ** ± ** 0.0926**	**0.4132 ** ± ** 0.0418**	**0.5512 ** ± ** 0.0111**	**0.5114 ** ± ** 0.0194**	**0.2914 ** ± ** 0.0154**
(w) GCN	0.2813 ± 0.0581	0.4672 ± 0.2247	0.6869 ± 0.0294	0.4887 ± 0.0450	0.2182 ± 0.0595	0.3451 ± 0.0343	0.4024 ± 0.0299	0.5392 ± 0.0646	0.4997 ± 0.0320	0.2523 ± 0.0237
(w) GNN	0.2573 ± 0.1021	0.5248 ± 0.0498	0.6593 ± 0.0827	0.4304 ± 0.0107	0.1754 ± 0.0118	0.3361 ± 0.0254	0.3005 ± 0.0227	0.4630 ± 0.0246	0.4796 ± 0.0743	0.2210 ± 0.0165
(w) Mean	0.2317 ± 0.0485	0.4349 ± 0.0450	0.6214 ± 0.0309	0.4343 ± 0.0636	0.2168 ± 0.0664	0.3135 ± 0.0615	0.3099 ± 0.0156	0.4661 ± 0.0161	0.4574 ± 0.0258	0.2214 ± 0.0112
**MSE** ↓	DLPFC	MBSP	HCC	HIC	MP	HS	HBCHD	MBHD	HL1	HL2
(w) GAT	**0.5987 ** ± ** 0.0432**	**0.8365 ** ± ** 0.0767**	**0.7829 ** ± ** 0.1272**	**1.0680 ** ± ** 0.0291**	**0.3651 ** ± ** 0.0440**	**0.3280 ** ± ** 0.0258**	**0.6467 ** ± ** 0.0344**	**0.5966 ** ± ** 0.0362**	**0.4857 ** ± ** 0.0047**	**0.3593 ** ± ** 0.0231**
(w) GCN	0.7042 ± 0.1023	1.2313 ± 0.2980	0.8432 ± 0.0816	1.1543 ± 0.1265	0.4618 ± 0.0520	0.4183 ± 0.0287	0.6830 ± 0.0434	0.7734 ± 0.1122	0.5114 ± 0.0324	0.4117 ± 0.0083
(w) GNN	0.6865 ± 0.1298	1.1048 ± 0.1275	0.9567 ± 0.4106	1.0700 ± 0.0421	0.5422 ± 0.0376	0.4654 ± 0.0695	1.0365 ± 0.0506	1.0191 ± 0.0256	0.6064 ± 0.0635	0.4910 ± 0.0149
(w) Mean	0.7254 ± 0.0703	1.0865 ± 0.1355	0.8677 ± 0.0606	1.1023 ± 0.1329	0.4122 ± 0.0557	0.4997 ± 0.0418	0.6967 ± 0.0214	0.6966 ± 0.0362	0.5821 ± 0.0156	0.4562 ± 0.0271
**MAE** ↓	DLPFC	MBSP	HCC	HIC	MP	HS	HBCHD	MBHD	HL1	HL2
(w) GAT	**0.6076 ** ± ** 0.0105**	**0.7056 ** ± ** 0.0331**	**0.6599 ** ± ** 0.0360**	**0.8106 ** ± ** 0.0409**	**0.2977 ** ± ** 0.0048**	**0.2921 ** ± ** 0.0191**	**0.4745 ** ± ** 0.0106**	**0.5233 ** ± ** 0.0088**	**0.4197 ** ± ** 0.0030**	**0.3230 ** ± ** 0.0071**
(w) GCN	0.6612 ± 0.0264	0.8481 ± 0.0958	0.6706 ± 0.0319	0.8293 ± 0.0459	0.3159 ± 0.0092	0.2933 ± 0.0147	0.5016 ± 0.0128	0.5466 ± 0.0355	0.4343 ± 0.0126	0.3412 ± 0.0050
(w) GNN	0.6589 ± 0.0537	0.7987 ± 0.0472	0.7252 ± 0.1312	0.8482 ± 0.0139	0.4070 ± 0.0097	0.3004 ± 0.0206	0.6359 ± 0.0131	0.5827 ± 0.0082	0.4731 ± 0.0215	0.3763 ± 0.0055
(w) Mean	0.6985 ± 0.0390	0.8024 ± 0.0499	0.7724 ± 0.0260	0.8704 ± 0.0582	0.3290 ± 0.0036	0.3208 ± 0.0080	0.6722 ± 0.0113	0.6421 ± 0.0131	0.4992 ± 0.0432	0.4002 ± 0.0142

The best result in each column is highlighted in bold.

## 4 Conclusions

STs still suffers from limited spot density and incomplete spatial coverage, making it difficult to accurately characterize fine-grained spatial gene expression patterns. In this work, we propose SpaBiT, a multimodal framework for spatial gene expression prediction and enhancement that integrates histological images with ST data. SpaBiT aggregates neighborhood information in the expression domain through GAT to capture local spatial structure, and extracts high-dimensional histology features from H&E images using a UNI-based encoder. These two modalities are fused by a bidirectional CA module and further processed by a Transformer, yielding expressive representations for spatial gene expression prediction. Experiments on multiple STs datasets demonstrate that SpaBiT consistently improves reconstruction accuracy over existing image-based and expression-based baselines, while better preserving tissue spatial organization. The SpaBiT-enhanced expression profiles also provide a more informative basis for downstream analyses such as spatial domain identification, tumor region delineation and functional enrichment, leading to clearer spatial patterns and biologically meaningful signals. Overall, SpaBiT offers an effective and flexible solution for multimodal spatial modeling and high-resolution exploration of spatial gene regulation and tissue microenvironments.

## Supplementary Material

btag443_Supplementary_Data

## Data Availability

The source code for this study is publicly available at GitHub (https://github.com/wenwenmin/SpaBiT). All datasets used in this study can be accessed and downloaded from Zenodo (https://zenodo.org/records/18044067).
