# Supplementary material for: SpaBiT: enhancing spatial transcriptomics resolution via bidirectional attention transformers
Source: Bioinformatics. 2026 Jul 2;42(7):btag443. doi: 10.1093/bioinformatics/btag443 (PMC13360274; doi:10.1093/bioinformatics/btag443)
Supplement: btag443_Supplementary_Data [file btag443_supplementary_data.pdf]

Supplementary Materials for

SpaBiT: Enhancing Spatial Transcriptomics Resolution via Bidirectional Attention Transformers

S1 Data Preprocess

Reconstruction of the pre-training dataset. To evaluate the model's imputation capability under realistic gene expression distributions and to establish effective training metrics, we construct, for each complete tissue section, a training set with "pseudo-missing" regions. Concretely, starting from the original spatial transcriptomics data with full expression profiles, we perform controlled downsampling to obtain a subset of spots used for training, while the remaining un-sampled spots are retained as prediction targets. In this way, we define a pre-training task with quantitatively comparable evaluation indices across methods.

More specifically, let the set of all spot coordinates within the tissue region of the original section be $\mathcal{C} = \{\mathbf{c}_i = (x_i, y_i)\}_{i=1}^{N}$, where N is the number of spots. To ensure that the downsampled region is spatially representative and that the structural properties of the original expression distribution are preserved, we dynamically set the grid step sizes Δx and Δy according to the layout of each section (square or hexagonal). We then perform regular sampling on this coordinate grid with strides of $2\Delta x$ and $2\Delta y$ along the horizontal and vertical directions, respectively, to obtain a low-resolution spot grid:

$$\mathcal{C}_{\mathrm{LR}} = \big\{(x, y) \,\big|\, x = x_{\min} + 2k\Delta x,\ y = y_{\min} + 2l\Delta y\big\} \tag{1}$$

where $x_{\min}$ and $y_{\min}$ are the minimum values among all spot coordinates, snapped to the nearest even integers, and $k, l \in \mathbb{Z}_{\geq 0}$.

Next, for each original coordinate $\mathbf{c}_i \in \mathcal{C}$, we determine whether it belongs to the low-resolution grid $\mathcal{C}_{\mathrm{LR}}$. If so, the corresponding spot is retained as a training sample; otherwise, it is removed from the training set and treated as a pseudo-missing target for imputation. This procedure yields a training

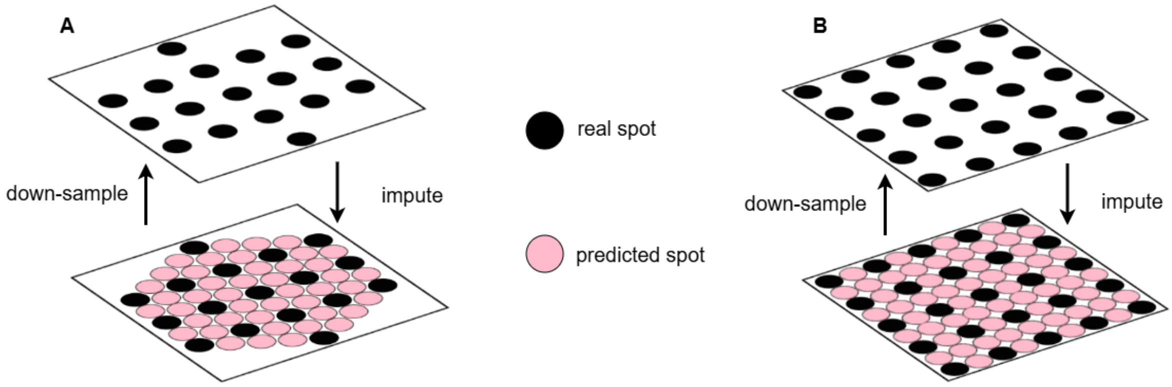

Fig S1: Downsampling-based reconstruction of the original data.

mask $\mathbf{m}_{\text{train}} \in \{0,1\}^N$, defined as

$$\mathbf{m}_{\text{train}}(i) = \begin{cases} 1, & \text{if } \mathbf{c}_i \in \mathcal{C}_{\text{LR}} \\ 0, & \text{otherwise} \end{cases} \tag{2}$$

This pre-training construction strategy simultaneously respects spatial structural constraints and the plausibility of expression distributions, providing a supervised training framework for the generative model. It also allows direct quantification of reconstruction error in the prediction regions, thereby enabling effective assessment of the generalization ability and spatial robustness of different imputation methods. The schematic illustration of the reconstruction procedure is shown in Fig. S1 , where panel A depicts hexagonal-grid downsampling for a standard Visium dataset with $\Delta x = 1, \Delta y = 2$, and panel B depicts rectangular-grid downsampling for the ST dataset with $\Delta x = 1, \Delta y = 1$.

S2 Model Parameters

The specific hyperparameter configurations and training settings employed for SpaBiT are summarized in Table S1.

Table S1: Hyperparameter configurations and training settings of SpaBiT.

**Hyperparameter / Setting**	**Value**
Number of neighbors for graph construction	6
GAT training epochs	3000
Number of GAT layers	2
Transformer training epochs	1000
Batch size	128
Learning rate	0.001
Number of Transformer layers	4
Optimization strategy	Adam with fixed learning rate

S3 Baseline Methods

The following section provides detailed descriptions of the baseline methods used for comparison in our experiments, covering various strategies for spatial gene expression prediction.

ST-Net (He et al., 2020): ST-Net is an image-driven convolutional neural network model that predicts spot-level gene expression directly from histology patches. It uses DenseNet-121 as the visual backbone to extract high-level image features and learns a mapping from these features to gene expression profiles for each spatial spot.

DeepSpaCE (Monjo et al., 2022): DeepSpaCE is another image-driven baseline that infers gene expression from local histology context. It adopts a VGG16-based architecture to encode image patches into visual embeddings and then predicts spot-wise expression levels through fully connected layers.

STAGE (Li et al., 2024): STAGE is a spatially supervised autoencoder that jointly models spot coordinates and gene expression. It encodes expression profiles into a low-dimensional latent space and reconstructs spatial coordinates as a supervision signal, using the learned latent representations to extrapolate gene expression at unmeasured locations.

HistoSGE (Shi et al., 2024): HistoSGE is a multimodal baseline that combines histology images and spatial positions. It uses the UNI model pretrained on large-scale pathology images to extract expressive visual features, and fuses them with positional encodings within a Transformer to predict spot-level gene expression profiles.

DIST (Zhao et al., 2023): DIST is an expression-only method that constructs high-resolution expression maps directly from low-resolution spatial transcriptomics data. It employs convolutional neural networks to learn latent structures in the expression space and generates finer-grained representations without using histological images or explicit spatial coordinates.

SpaViT (Min et al., 2025): SpaViT is a Vision Transformer-based baseline that treats low-resolution expression vectors as sequence inputs. By applying self-attention to capture long-range dependencies within the expression profiles, it models high-resolution gene expression patterns solely from transcriptomic information, without any auxiliary imaging modality.

NN: NN interpolation is a distance-based baseline that assigns each high-resolution location the gene expression profile of the nearest observed spot in the original spatial transcriptomics data. It performs piecewise-constant extrapolation in the spatial coordinate space without using histology or other auxiliary information.

Linear: Linear interpolation estimates gene expression at an unmeasured location as a weighted average of nearby spots, with weights decreasing linearly with distance (equivalently, bilinear interpolation on a regular grid). It relies solely on spatial coordinates and is applied independently to each gene.

Cubic: Cubic interpolation reconstructs high-resolution expression maps using bicubic interpolation over the spatial grid. It generates smooth expression surfaces for each gene based only on the spatial arrangement of observed spots, without incorporating histological features or prior structures.

S4 Evaluation

1. Pearson Correlation Coefficient (PCC)

PCC evaluates the linear relationship between predicted and ground-truth values. A value closer to 1 indicates stronger correlation and superior performance:

$$\text{PCC} = \frac{\text{Cov}(X_{obs}, X_{pred})}{\sqrt{\text{Var}(X_{obs}) \cdot \text{Var}(X_{pred})}} \tag{3}$$

where X_{obs} and X_{pred} represent the observed ground-truth and predicted gene expression values, respectively.

2. Mean Squared Error (MSE)

MSE measures the average squared difference between predictions and ground truth, providing sensitivity to large outliers:

$$\text{MSE} = \frac{1}{N}\sum_{i=1}^{N}(X_{obs,i} - X_{pred,i})^2 \tag{4}$$

3. Mean Absolute Error (MAE)

MAE assesses the average error magnitude without considering direction, reflecting overall prediction deviation:

$$\text{MAE} = \frac{1}{N}\sum_{i=1}^{N}|X_{obs,i} - X_{pred,i}| \tag{5}$$

S5 Gene-wise Jensen-Shannon Divergence Comparison Results

To further validate the performance of SpaBiT, we conducted a distribution alignment benchmark against several state-of-the-art deep learning baselines for spatial transcriptomics prediction, including STNet, DeepSpaCE, SpaViT, STAGE, and DIST. We utilized the Jensen-Shannon (JS) divergence to quantify the discrepancy between the predicted and ground-truth spatial gene expression profiles across the 12 DLPFC tissue slices.

As shown in Fig. S2, SpaBiT consistently achieves the lowest JS divergence among all compared methods across all evaluation slices. This substantial margin demonstrates that our framework provides a closer alignment with the actual tissue expression data, showcasing its strength in accurately capturing the global transcriptomic distributions and spatial heterogeneity.

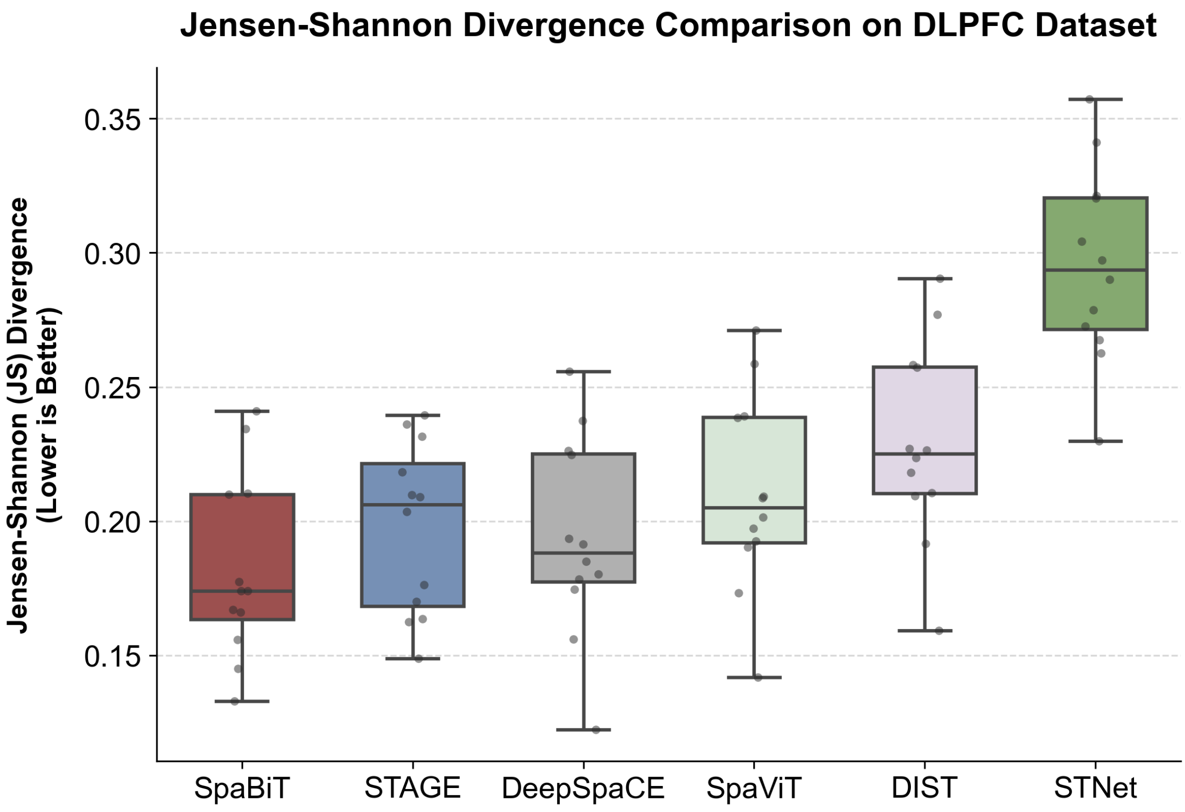

Fig S2: Boxplots show the distribution of sample-specific Jensen-Shannon (JS) divergence values between predicted and ground-truth spatial gene expression maps for different methods across DLPFC 12 tissue slices.

S6 Supplementary Results

To further evaluate the robustness and generalization of SpaBiT across diverse biological contexts, we provide additional visualization results for three datasets: HBCHD, HL1, and HL2. Specifically, Fig. S3, Fig. S4, and Fig. S5 illustrate the spatial expression patterns of selected marker genes in these respective datasets, comparing the ground truth with the reconstruction results from SpaBiT and baseline methods. These visualizations consistently demonstrate that SpaBiT effectively restores both global spatial trends and fine-grained local variations, maintaining high fidelity to the original biological structures across all tested datasets.

S7 Ablation studies

To rigorously evaluate the necessity of the proposed bidirectional cross-attention mechanism for multi-modal integration, we further extended our ablation experiments by entirely removing the bidirectional attention module and replacing it with simpler, non-attention fusion baselines. Specifically, we implemented two classic alternative strategies for comparison: (1) **Feature Concatenation**, where the visual morphology features and spatial neighborhood topology features are directly concatenated along the channel dimension followed by a linear projection, and (2) **Weighted Element-wise Fusion**, which

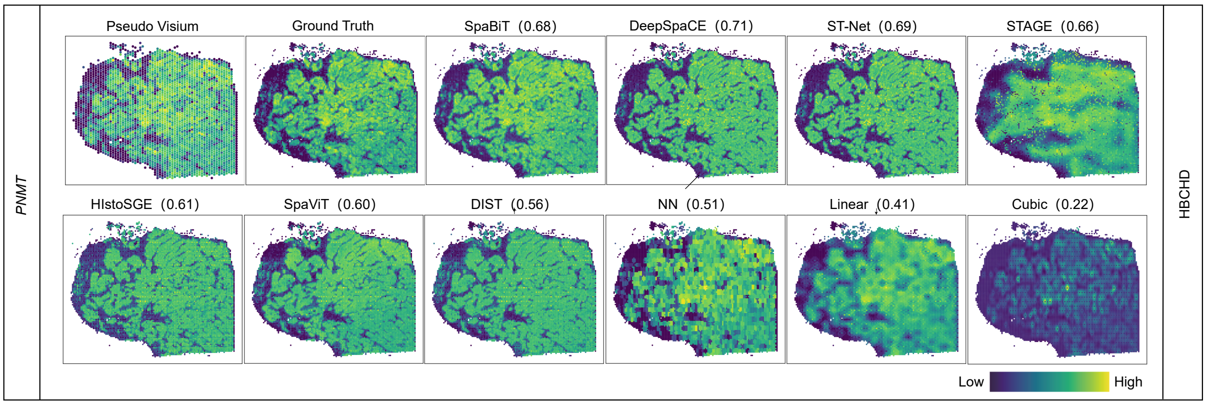

Fig S3: Spatial expression of *PNMT* on the HBCHD dataset. Panels (left to right) display the pseudo Visium input, ground truth, and reconstructions by SpaBiT (ours) and compared methods (DeepSpaCE, ST-Net, STAGE, HistoSGE, SpaViT, DIST, and NN/Linear/Cubic interpolations). Colors represent normalized expression levels (purple to yellow). PCC values relative to ground truth are provided in parentheses.

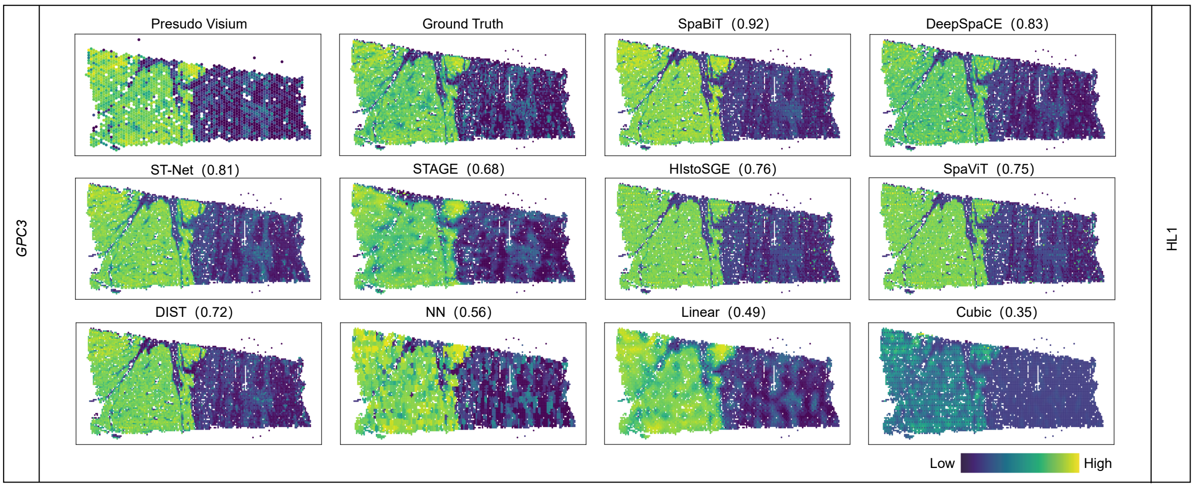

Fig S4: Spatial expression of *GPC3* on the HL1 dataset. Panels (left to right) show pseudo Visium input, ground truth, and reconstructions by SpaBiT (ours) and compared methods (DeepSpaCE, ST-Net, STAGE, HistoSGE, SpaViT, DIST, and NN/Linear/Cubic interpolations). Colors denote normalized expression (purple to yellow). PCC values are provided in parentheses.

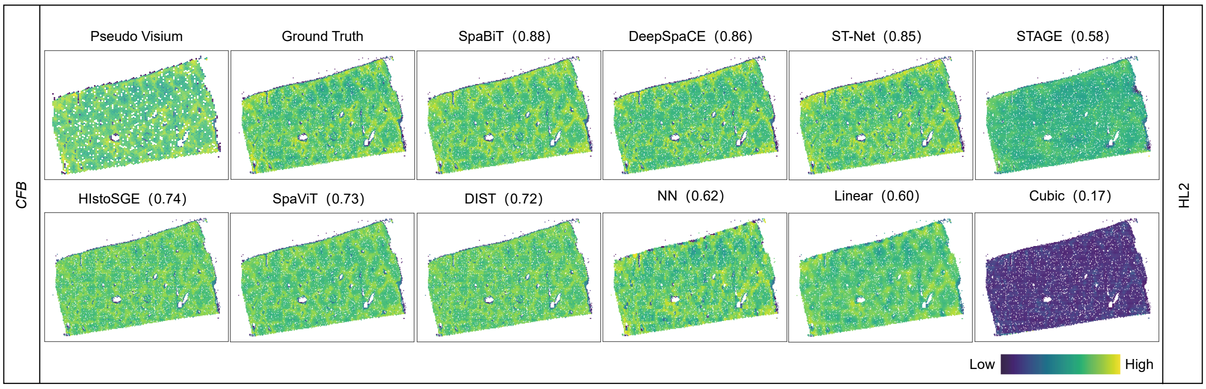

Fig S5: Spatial expression of *CFB* on the HL2 dataset. Panels (left to right) show pseudo Visium input, ground truth, and reconstructions by SpaBiT (ours) and compared methods (DeepSpaCE, ST-Net, STAGE, HistoSGE, SpaViT, DIST, and NN/Linear/Cubic interpolations). Colors denote normalized expression (purple to yellow). PCC values are provided in parentheses.

linearly scales and sums the two heterogeneous feature representations.

These additional ablation experiments were evaluated on the DLPFC dataset. As illustrated in in Fig.

S6, both simple fusion alternatives exhibit a noticeable performance degradation across all key evaluation metrics compared to the full SpaBiT framework. These quantitative results empirically demonstrate that simple linear combinations fail to adequately reconcile the cross-modal heterogeneity between visual context and spatial topology, thereby solidifying the architectural superiority and indispensability of our bidirectional cross-attention design.

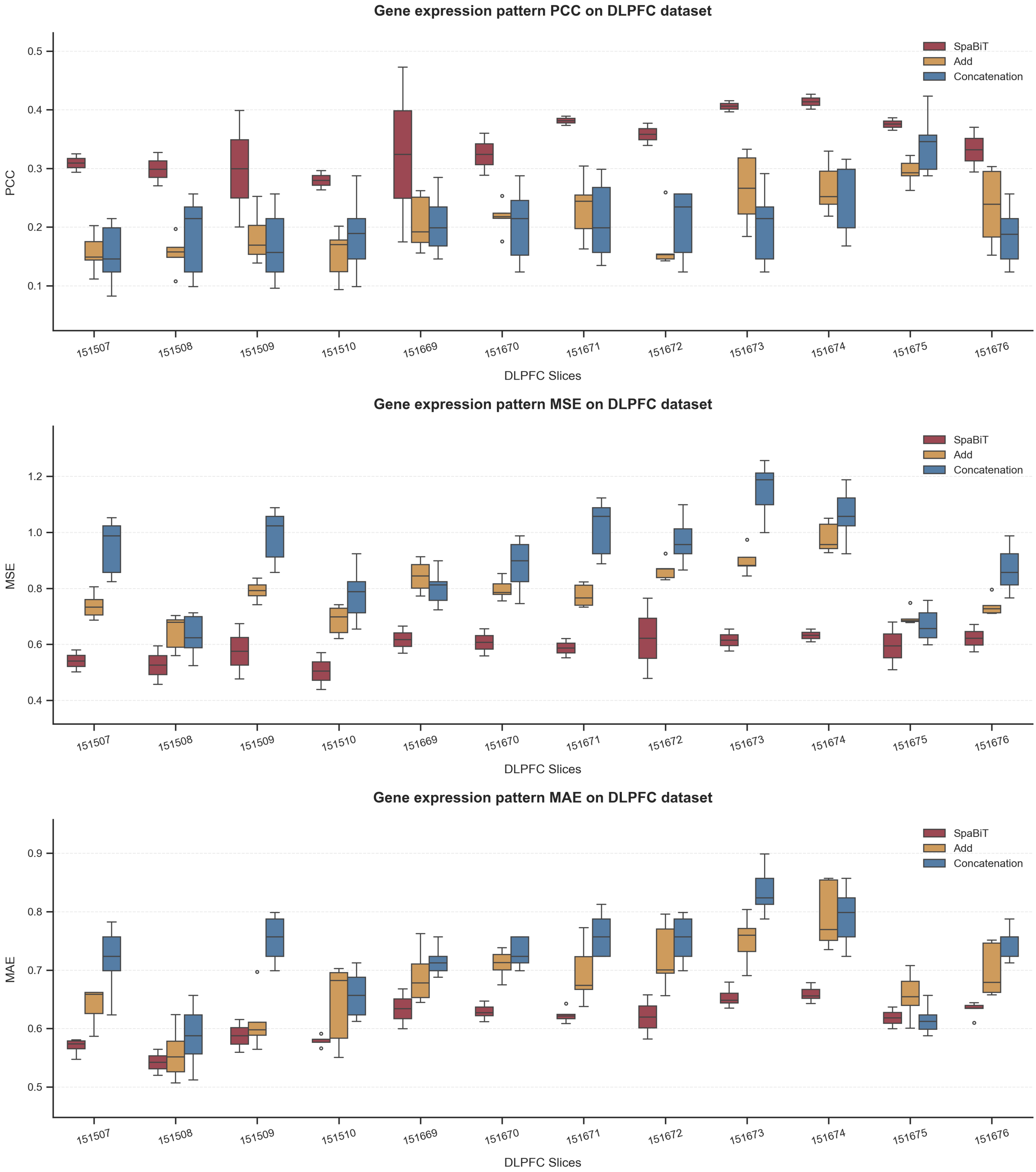

Fig S6: Ablation performance comparison of multi-modal fusion strategies on the DLPFC dataset. The box plots illustrate sample-specific performance across 12 tissue slices evaluated via three key metrics: Pearson Correlation Coefficient (PCC), Mean Squared Error (MSE), and Mean Absolute Error (MAE). SpaBiT (ours, bidirectional cross-attention) consistently outperforms the two non-attention baseline alternatives, Add (element-wise addition) and Concatenation, achieving superior prediction accuracy and robustness.

S8 Parameter Ablation on Spatial Graph Construction

To investigate the impact of the number of neighbors K utilized during spatial graph construction, we conducted a parametric ablation study on DLPFC slices 151507 and 151508. We varied the value of K from 3 to 9 and evaluated the corresponding performance using PCC, MSE, and MAE. As illustrated in Fig. S7, the model performance exhibits a clear response to the change of K. For both tissue slices, the Pearson Correlation Coefficient (PCC) peaks at $K = 6$, while the error metrics (MSE and MAE) concurrently reach their lower bounds around this region. This consistent trend demonstrates that a moderate number of neighbors provides an optimal graph topology, successfully capturing relevant spatial contexts without introducing excessive noise from distant spots. Based on these empirical findings, $K = 6$ was selected as the default hyperparameter for graph construction in our framework.

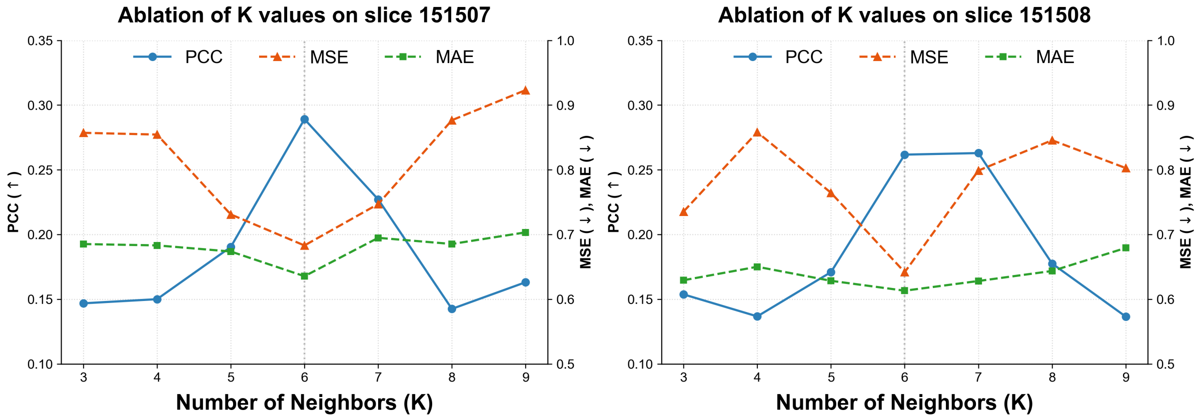

Fig S7: **Ablation study on the number of neighbors (K) in spatial graph construction.** The line plots illustrate the model performance under varying K values across DLPFC slices 151507 (left) and 151508 (right). The left y-axis denotes the Pearson Correlation Coefficient, while the right y-axis represents the error metrics (MSE and MAE).

References

He, B., Bergenstråhle, L., Stenbeck, L., et al. (2020). Integrating spatial gene expression and breast tumour morphology via deep learning. *Nature Biomedical Engineering*, 4(8):827–834.

Li, S., Gai, K., Dong, K., Zhang, Y., and Zhang, S. (2024). High-density generation of spatial transcriptomics with STAGE. *Nucleic Acids Research*, 52(9):4843–4856.

Min, W., Xue, S., Zhu, F., Xu, T., Wang, C., and Xie, H.-B. (2025). SpaViT: self-supervised prediction of high-resolution spatial transcriptomics with vision transformer. *Tsinghua Science and Technology*.

Monjo, T., Koido, M., Nagasawa, S., Suzuki, Y., and Kamatani, Y. (2022). Efficient prediction of a spatial transcriptomics profile better characterizes breast cancer tissue sections without costly experimentation. *Scientific Reports*, 12(1):4133–4145.

Shi, Z., Xue, S., Zhu, F., and Min, W. (2024). High-resolution spatial transcriptomics from histology images using HisToSGE. In *Proceedings of the 2024 IEEE International Conference on Bioinformatics and Biomedicine (BIBM)*, pages 2402–2407.

Zhao, Y., Wang, K., and Hu, G. (2023). DIST: spatial transcriptomics enhancement using deep learning. *Briefings in Bioinformatics*, 24(2):bbad013.
